# Genome Analysis of *Staphylococcus capitis* TE8 Reveals Repertoire of Antimicrobial Peptides and Adaptation Strategies for Growth on Human Skin

**DOI:** 10.1038/s41598-017-11020-7

**Published:** 2017-09-05

**Authors:** Rohit Kumar, Pramod Kumar Jangir, Jhumki Das, Bhupesh Taneja, Rakesh Sharma

**Affiliations:** 1 CSIR-Institute of Genomics and Integrative Biology, Council of Scientific and Industrial Research (CSIR), New Delhi, India; 2 Academy of Scientific and Innovative Research (AcSIR), New Delhi, India; 3 Food Corporation of India, Ludhiana, India; 4 Present Address: Synthetic and Systems Biology Unit, Institute of Biochemistry, Biological Research Centre of the Hungarian Academy of Sciences, Szeged, 6726 Hungary

## Abstract

*Staphylococcus capitis* TE8 was isolated from skin surface of a healthy human foot, and exhibited a strong antibacterial activity against Gram-positive bacteria, including *Staphylococcus aureus*. Whole genome sequence of *S. capitis* TE8 was obtained by shotgun and paired-end pyrosequencing with a coverage of 109-fold. The draft genome contains 2,516,639 bp in 8 scaffolds with 209 total contigs. The genome contains 2319 protein coding sequences, 58 tRNA and 3 rRNA. Genome sequence analysis revealed 4 distinct gene loci with the ability to encode antimicrobial peptides: (i) an epidermicin gene cluster; (ii) a gallidermin gene cluster; (iii) a gene cluster encoding six phenol soluble modulin (PSM) β-type peptides (PSMβ1-β6) and (iv) an additional gene that belonged to PSMβ family and encoded a 44 residues long peptide, HTP2388. Synthetic peptides with sequence identical to seven PSMβ-like peptides i.e. PSMβ1-β6 and peptide HTP2388 showed antibacterial activity. Genome sequence also revealed genes for adhesins, intracellular adhesins, osmoadaptation, oxidative and acid stress tolerance possibly responsible for initial attachment, colonization and survival of *S. capitis* TE8 on human skin. Comparative genome analysis revealed presence of a gamut of genes in *S. capitis* strains in comparison to *Staphylococcus epidermidis* and *Staphylococcus caprae* indicating towards their possible role in better adaptation and survival on human skin.

## Introduction

The skin is the outermost covering of human body and provides protection from numerous invading agents and toxic substances^1^. Human skin is a host to a large number of microbes, which exhibit distinct topographical niches and significant diversity depending on the skin site^2, 3^. While a general commensal role *vs*. a mutually beneficial symbiotic relationship with the host cells is not clear for the microbial populations, several of these microbes are known to protect against colonisation and invasion by pathogenic or harmful organisms in different ways^4–6^. For instance, resident bacteria on the skin may secrete antimicrobial agents and thereby help in maintaining a safe environment on the skin^7^. Recently, the large diversity of microbial populations in the human microbiome has been found to encode biosynthetic gene clusters that offer a rich potential for bioactive compounds with an ability to modulate host-microbe interactions^8, 9^. At the same time, the human microbiota offers an enormous resource to look for new antimicrobial agents^4^.

Microbe-derived natural products have been the classical source of antibiotics and antimicrobial compounds^10^. The emergence of antimicrobial resistance in bacteria over the last few decades mandates an urgent need to search for new sources of therapeutic agents. The skin microbiota is envisaged to harbour a large repertoire of antimicrobial agents. Staphylococci are common colonizers of human skin; represented as 3^rd^ largest genera out of the 205 genera identified in human skin microbiome using the 16S rRNA gene phylotyping^2^. *S. epidermidis* and *Staphylococcus hominis, Staphylococcus capitis*, *Staphylococcus caprae*, *Staphylococcus auricularis*, *Staphylococcus warneri, Staphylococcus aureus* and *Staphylococcus haemolyticus* are among the commonly isolated species from human skin^11^. They are abundant at sebaceous sites and also represented at the moist sites of human skin^2^. These staphylococci play an important role in skin protection by their ability to reduce the pathogen load on the skin surface and also maintain community structure on the skin surface effectively^12, 13^. For instance, *S. epidermidis* has the ability to inhibit *S. aureus* colonization in nasal cavities by secreting a serine protease, Esp^5^ while *S. lugdunensis* impairs *S. aureus* colonization by the production of a bioactive compound, Lugdunin^4^.

In this study, we isolated a bacterium *Staphylococcus capitis* TE8 from the skin surface of a healthy human foot, which exhibited a strong antibacterial activity against Gram positive bacteria, including *S. aureus*. The genes encoding for antimicrobial peptides were identified using a whole genome sequencing approach. Whole genome sequencing of *S. capitis* TE8 enabled us to identify 4 distinct gene loci with the ability to encode antimicrobial peptides: (i) an epidermicin gene cluster; (ii) a gallidermin gene cluster; (iii) a gene cluster encoding six phenol soluble modulin (PSM) β-type peptides (PSMβ1-β6) and (iv) an additional gene that belonged to PSMβ family and encoded a 44 residues long peptide HTP2388. We further demonstrate that the antibacterial activity of *S. capitis* TE8 was associated with all the seven PSMβ-like peptides i.e. six PSMβ peptides and peptide HTP2388. This work adds to the growing understanding that the microbial community in the skin may be a rich source of novel antimicrobial molecules that provide a secure environment both to the microbe from competing microbes and to the human host against skin infections from other pathogens.

## Results and Discussion

### Identification of human skin isolate TE8 with antimicrobial activity

**Figure 1:**
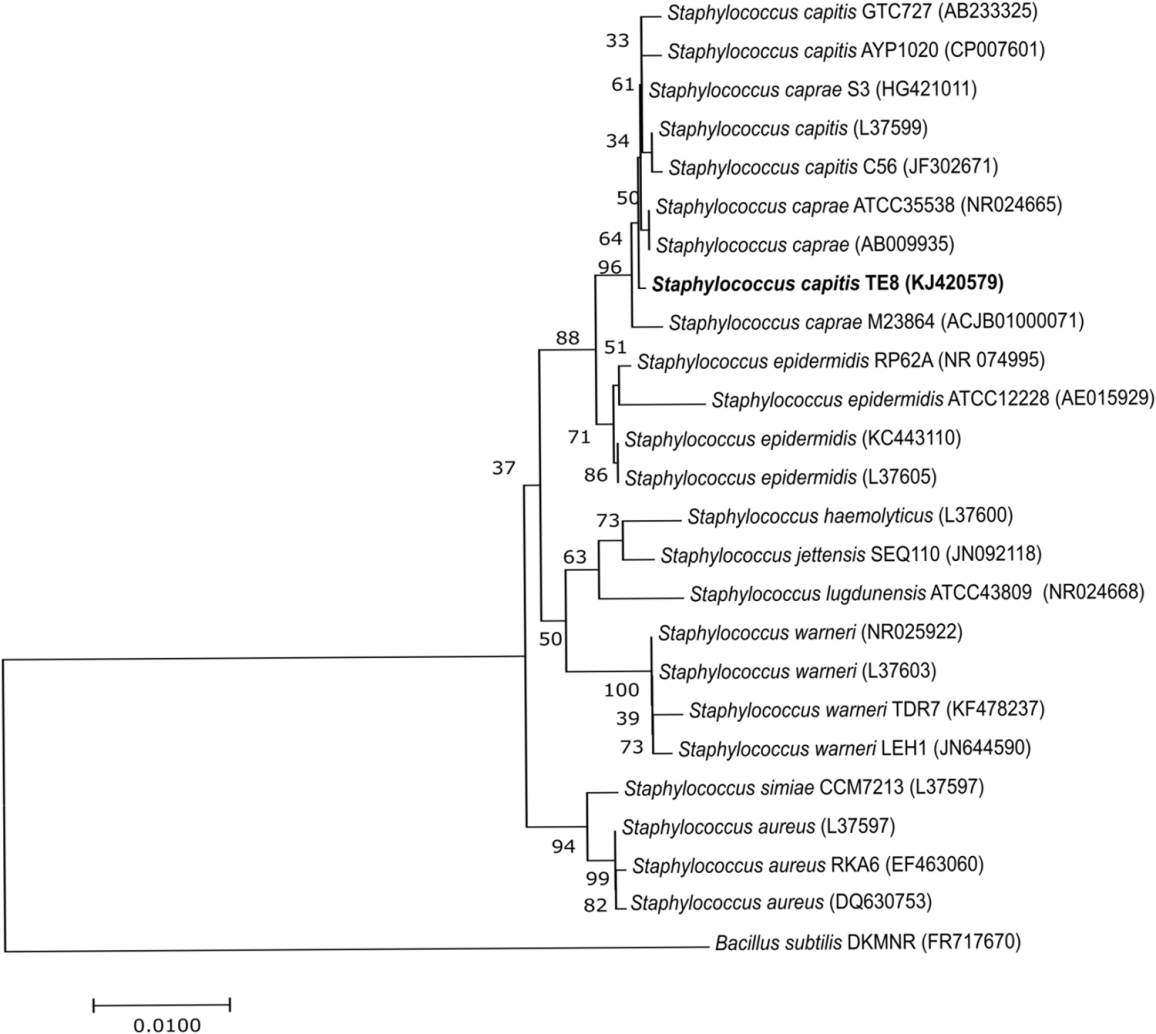
16S rRNA phylogenetic analysis of *Staphylococcus capitis* TE8 with related *Staphylococcus* species by neighbor-joining method. Phylogenetic tree was constructed by MEGA7 software and *Bacillus subtilis* strain DKMNR was used as the outgroup.

The bacterial isolate TE8 was isolated from the skin surface of the foot of a healthy individual and identified by 16S rRNA gene sequence analysis. The amplified region of the 16S rRNA gene of isolate TE8 showed 99.86% sequence identity with *rrs* of both *S. caprae* ATCC 35538 (T) and *S. capitis* subsp*. capitis* ATCC 27840 (T) (Fig. 1). In-silico DNA-DNA hybridization (DDH) by genome to genome distance calculator revealed that *S. capitis* AYP1020, with a score of 88.5, is the closest species followed by 26.4 for *S. caprae* and 22.5 for *S. epidermidis*, indicating that isolate TE8 may be a new strain of *S. capitis*. The strain was hence designated as *S. capitis* TE8.

**Figure 2:**
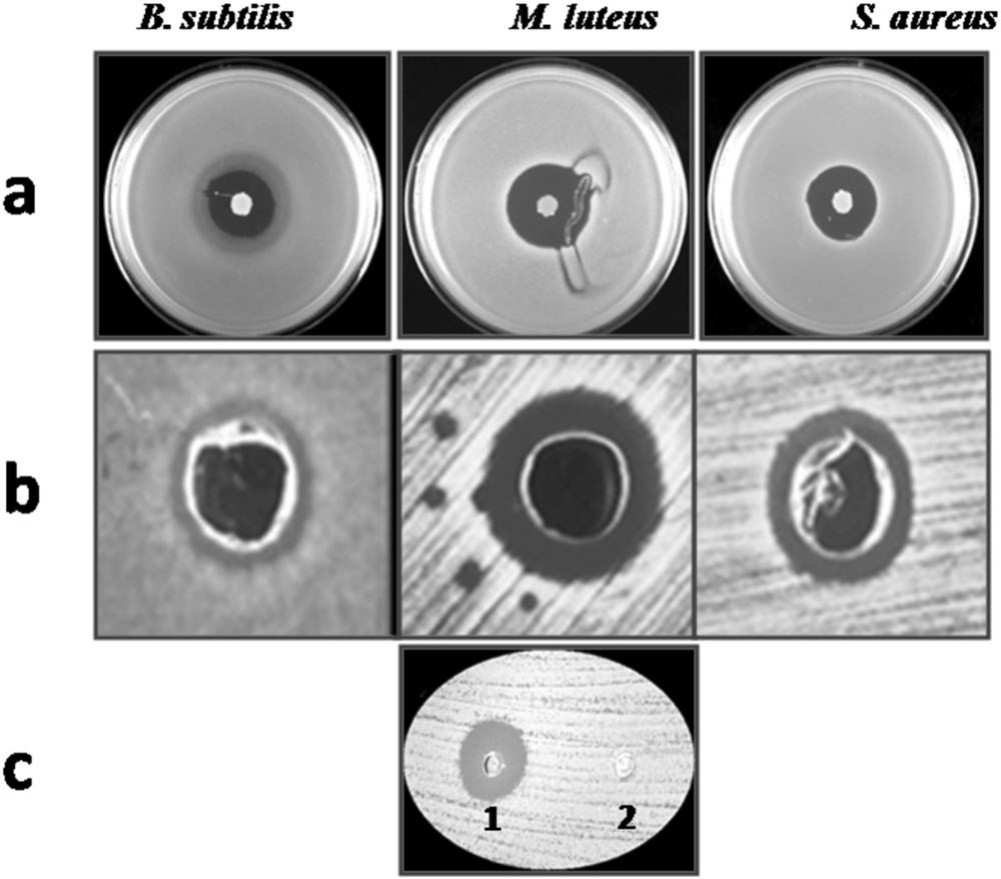
Antibacterial activity of *Staphylococcus capitis* TE8. (**a**) Antibacterial activity of *S. capitis* TE8 against *Bacillus subtilis*, *Micrococcus luteus* and *Staphylococcus aureus*. (**b**) Antibacterial activity of crude 1-butanol extracts of isolate TE8 against *B. subtilis*, *M. luteus* and *S. aureus*. (**c**) Proteinase K treatment effect on 1-butanol extract: 1- Crude extract, 2 - Proteinase K treated extract on *M. luteus*.

*S. capitis* TE8 showed antibacterial activity against Gram positive organisms *Micrococcus luteus, S. aureus* and *Bacillus subtilis* (Fig. 2a) but not against Gram negative strains *i.e. Escherichia coli*, and *Pseudomonas aeruginosa* or fungus *Candida glabrata* (data not shown). The antibacterial activity of the 1-butanol extract was examined against the Gram positive test strains, namely, *M. luteus, S. aureus, and B. subtilis* (Fig. 2b)*. M. luteus* was found to be relatively more susceptible to the extract of *S. capitis* TE8, showing a clear and wider zone of inhibition. The extract also exhibited antibacterial activity against *B. subtilis* and *S. aureus*. Proteinase K treatment of the extract resulted in complete loss of antimicrobial activity on *M. luteus* (Fig. 2c), suggesting the antimicrobial agent to be proteinaceous in nature. Various *Staphylococcus* species have also been shown to produce proteins or peptides that inhibit the growth of *S. aureus* and other Gram positive organisms, e.g. *S. gallinarum* produces gallidermin^14^, *S. epidermidis* 224 produces epidermicin N101^15^, *S. warneri* produces warnerin^16^ and *S. lugdunensis* produces Lugdunin^4^; likewise, *S. capitis* TE8 may encode protein/peptides with antimicrobial properties.

### Genome analysis of *Staphylococcus capitis* TE8

**Figure 3:**
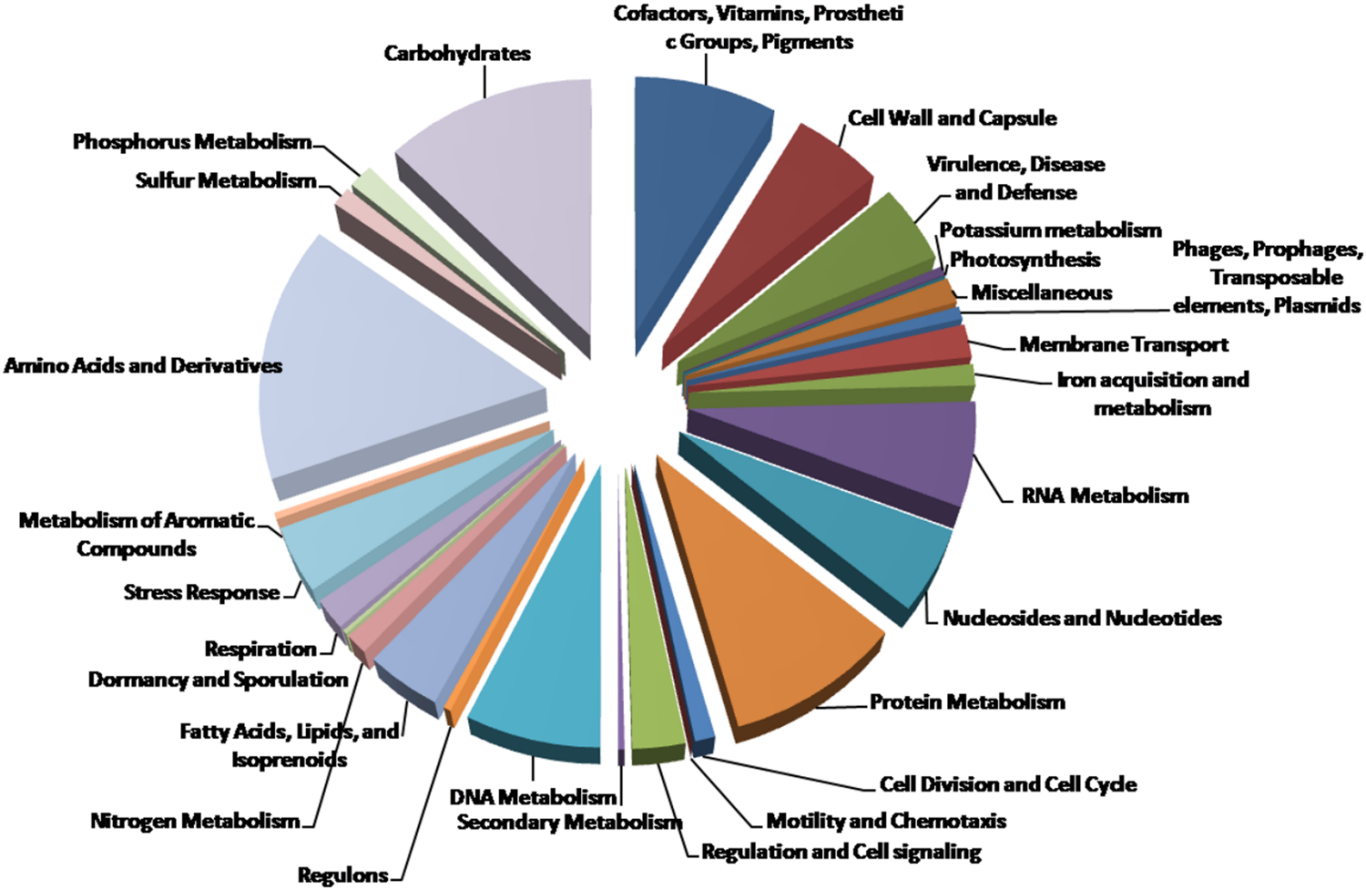
Functional categories of subsystem features predicted in the *Staphylococcus capitis* TE8 genome.

Whole genome sequencing of *S. capitis* TE8 yielded a total of 1,465,735 reads (105,156 shotgun reads and 1,360,579 paired-end reads). The estimated genome size of *S. capitis* TE8 is 2.8 Mb with a coverage of 109-fold. The draft genome contains 2,516,639 bp in 8 scaffolds with 209 total contigs. Genome annotation on the NCBI PGAP annotation pipeline identified 2319 protein coding sequences, 58 tRNA and 3 rRNA in the genome (Table 1). The predicted protein coding sequences were annotated and assigned to putative biological functions (Fig. 3). The genome possesses 8 genomic islands (Supplemental Fig. S1) and the genes present in them are included in Supplemental Table S1. Most of the islands harbor genes encoding for hypothetical proteins and one of them appears to be a prophage. Presence of genes encoding for antimicrobial peptides and nutritional stress resistance proteins reflect evolutionary advantage that the predicted GIs might confer on *S. capitis* TE8.

**Table 1:** General features of *Staphylococcus capitis* TE8 genome.

Total Length (bp)	2,516,639
G+C content (%)	32.8
Number of predicted coding sequences (cds)	2421
Pseudogenes	41
Number of predicted proteins	2319
tRNA	58
rRNA	3

**Figure 4:**
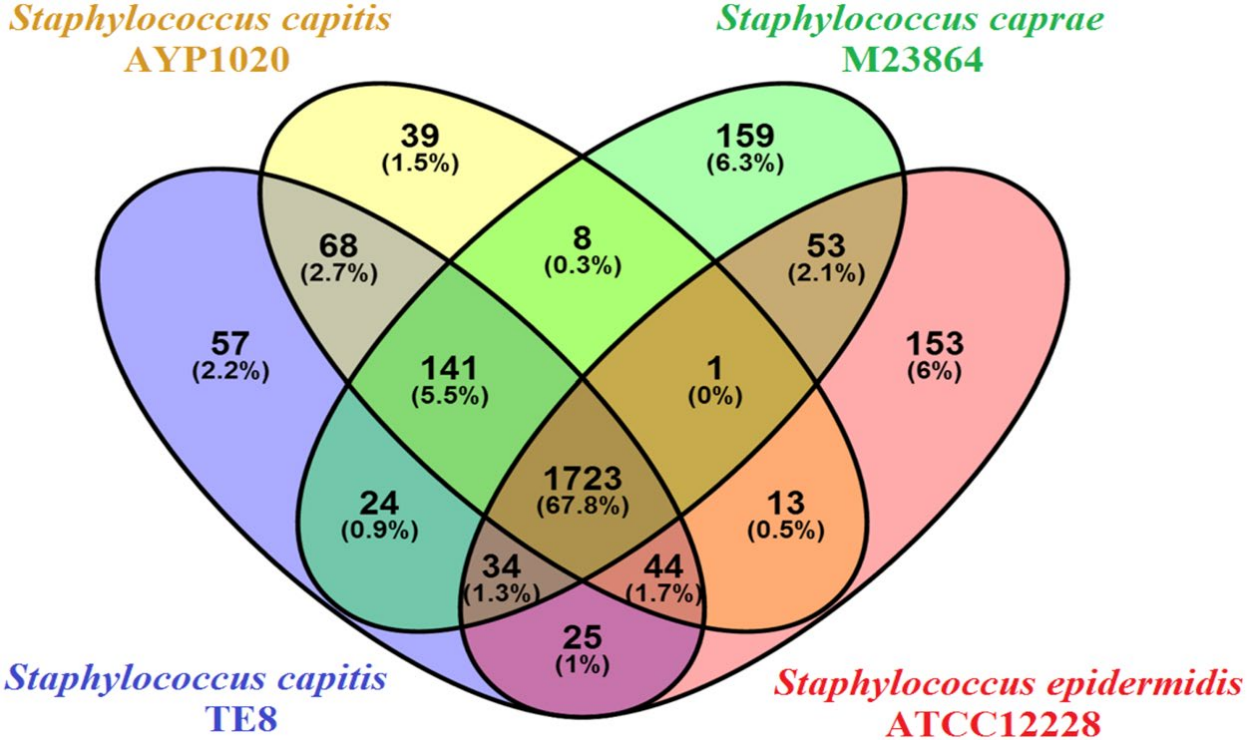
Venn diagram showing the core and strain specific proteins in four *Staphylococcus* spp. OrthoMCL v2.0.9 was used to generate clusters of orthologous proteins, which are indicated by overlapping regions.

Comparative genome analysis using OrthoMCL revealed clustering of *S. capitis* TE8 CDSs in 2116 families, out of which 1723 (67.8%) were shared with other three bacteria, *S. capitis* AYP1020*, S. caprae* and *S. epidermidis* (Fig. 4, Supplemental Fig. S2). The remaining 393 orthologous groups were defined the accessory genome, including 57 unique genes present only in *S. capitis* TE8. Comparison between genomes of these strains using RAST (Supplemental Table S2) revealed that *S. capitis* TE8 shared a majority of subsystems with *S. capitis* AYP1020 but also have few distinct differences. *S. capitis* TE8 contains genes for polyhydroxybutyrate metabolism, inorganic sulphur assimilation and antibiotic & toxic compounds resistance genes, which are absent in *S. capitis* AYP1020. While genes for some important subsystems were absent in *S. capitis* TE8 genome but were present in *S. capitis* AYP1020, such as genes in bacterial check point control, cell division and cell control, lysine biosynthesis, carotenoids, isoprenoid biosynthesis and polyprenyldiphosphate biosynthesis.

### *Staphylococcus capitis* TE8 encodes distinct antimicrobial peptides

The annotated genome of *S. capitis* TE8 was analysed using different servers viz. AntiSMASH, BAGEL3 and RAST to identify the genes which may encode antibacterial proteins/peptides in the genome. Genome analysis revealed (i) a gene cluster for an epidermicin-type protein, (ii) a gene cluster for gallidermin-type protein, (iii) a PSMβ-type peptide gene cluster of six genes and another (iv) PSMβ-related peptide, HTP2388.

### Epidermicin-like antimicrobial peptide

**Figure 5:**
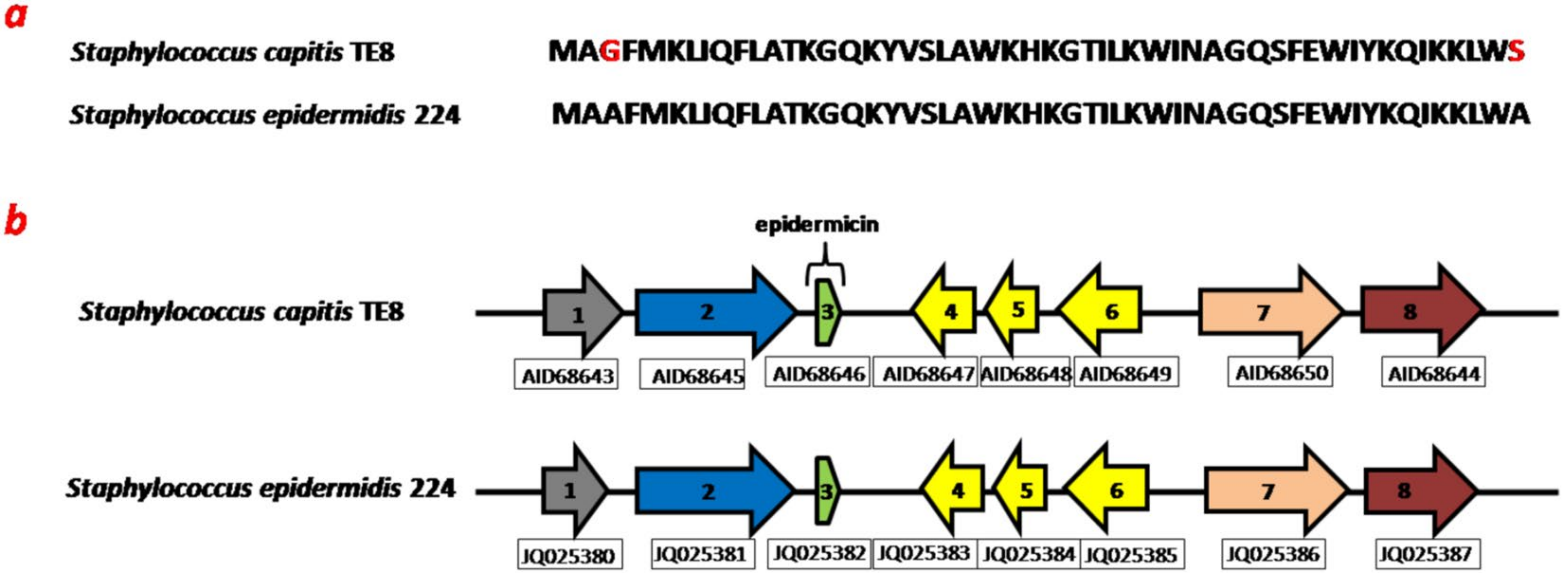
Genetic organization and sequence of putative epidermicin locus of *Staphylococcus capitis* TE8 compared to epidermicin N101. Epidermicin gene cluster arrangement of *S. capitis* TE8 and *S. epidermidis* 224. 1- YbdS like protein, 2- YbdT like protein, 3- epidermicin, 4, 5, 6- hypothetical proteins, 7- RND efflux like protein, 8- ABC transport ATP-binding protein. Accession no JQ025380 - JQ025387 and KJ702950 for epidermicin gene cluster of *S. epidermidis* 224 and nucleotide sequence (having epidermicin gene cluster) of *S. capitis* TE8, respectively.

A gene encoding a 51-residue long epidermicin-like protein was identified in the genome of *S. capitis* TE8 using RAST server and BAGEL3 software. Epidermicins are ribosomally synthesized antibacterial proteins/peptides of type II bacteriocin family. Most significant homology was found with epidermicin N101 (accession number AFD03077) of *S. epidermidis* 224^15^ with 96% sequence identity and only two residue variations. Predicted epidermicin of *S. capitis* TE8 contains glycine and serine at 3^rd^ and 51^st^ positions instead of alanine at corresponding positions for epidermicin N101 of *S. epidermidis* 224 (Fig. 5a). The genetic organization of the epidermicin cluster of *S. capitis* TE8 is also similar to that of epidermicin N101 in *S. epidermidis* 224 with a similar arrangement of neighboring genes (Fig. 5b). It is present on a genomic island of length 6307 bp (Supplemental Table S1). Epidermicins are potent antibacterial agents that display activity against Gram positive pathogens like *S. epidermidis*, methicillin resistant *S. aureus* (MRSA) isolates, and vancomycin-resistant enterococci (VRE)^15^. High sequence similarity and similar gene organization of putative epidermicin from *S. capitis* TE8 with epidermicin of *S. epidermidis* 224 indicates that epidermicin of *S. capitis* TE8 is likely to have similar potent antibacterial activity.

### Gallidermin biosynthesis genes cluster

**Figure 6:**
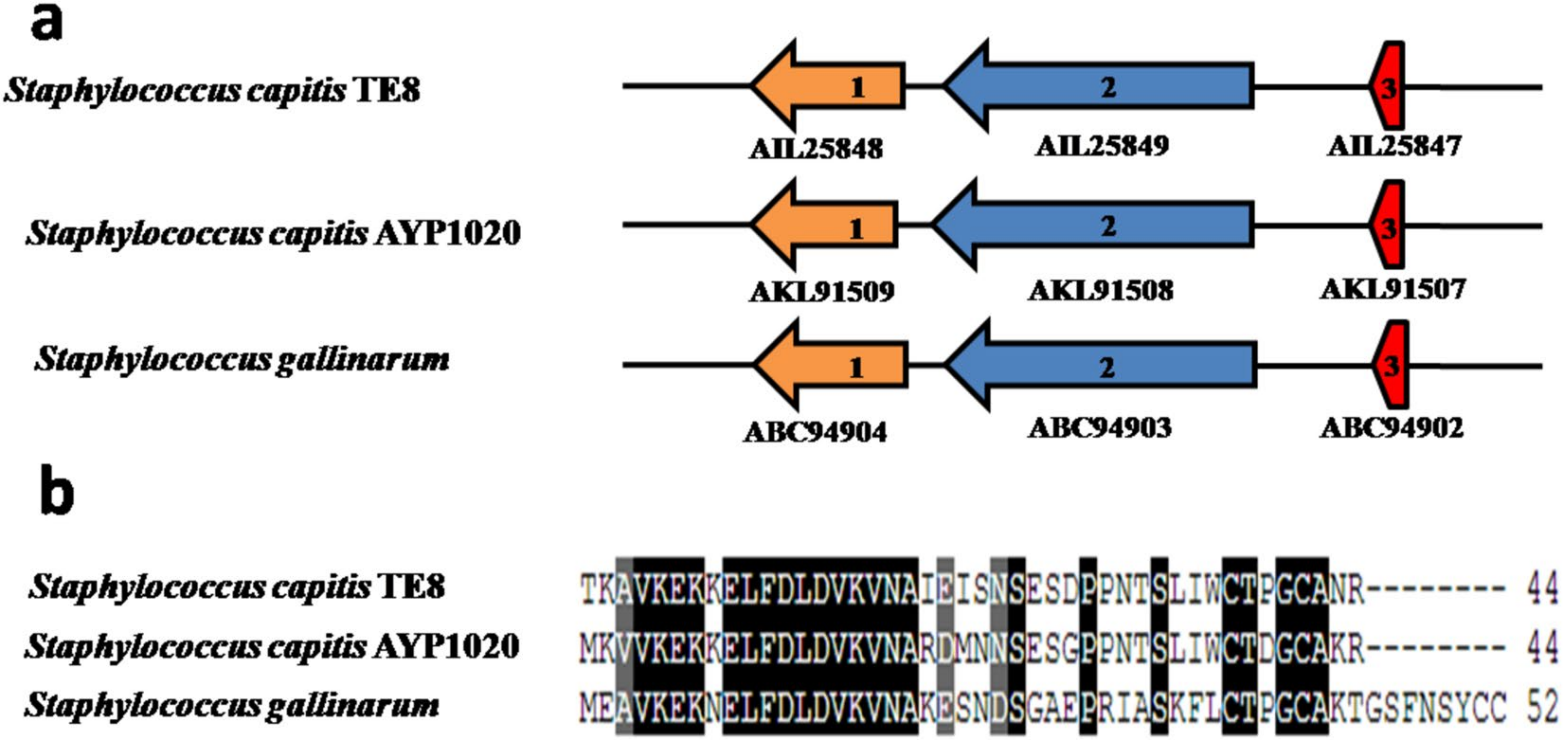
Genetic organization and sequence alignment of putative gallidermin locus of *Staphylococcus capitis* TE8. (**a**) Putative gallidermin gene cluster arrangement of *S. capitis* TE8 along with *S. capitis* AYP1020 and *S. gallinarum*. 1- LanC cyclase, 2- LanB dehydratase, 3- gallidermin. (**b**) Sequence alignment of gallidermin protein of *S. capitis* TE8, *S. capitis* AYP1020 and *S. gallinarum*. Arrow indicates the start site predicted by antiSMASH.

A putative gallidermin-encoding gene in the genome of *S. capitis* TE8 was identified using antiSMASH server (Fig. 6a). Gallidermins are lanthionine-containing peptide antibiotics having inhibitory activity against propionibacteria^14^. Gallidermins consist of a leader peptide and an active core region with antimicrobial activity. Previously characterized gallidermin of *S. gallinarum* was identified as a 52 residue-long peptide with 30 residues associated with leader peptide and 22 residues with the core peptide^17^. The predicted gallidermin of *S. capitis* TE8 as identified by antiSMASH server was manually curated to a 44-residue long peptide with ACG as the possible start codon of the gene encoding the gallidermin (Fig. 6b). Blastp search of this putative gallidermin-like protein shows homology with a hypothetical protein (98% identity; WP_016898795) encoded by *S. capitis*, a gallidermin-type hypothetical peptide (81% identity; WP_002469996) encoded by *S. capitis*, gallidermin (63% identity; P21838) of *S. gallinarum* ^17^ and epidermin (59% identity; P08136) of *S. epidermidis* ^18^, suggesting similar antimicrobial functions for the *S. capitis* TE8 encoded peptide.

### Cluster of PSMβ peptides (PSMβ1-β6) and peptide HTP2388

**Figure 7:**
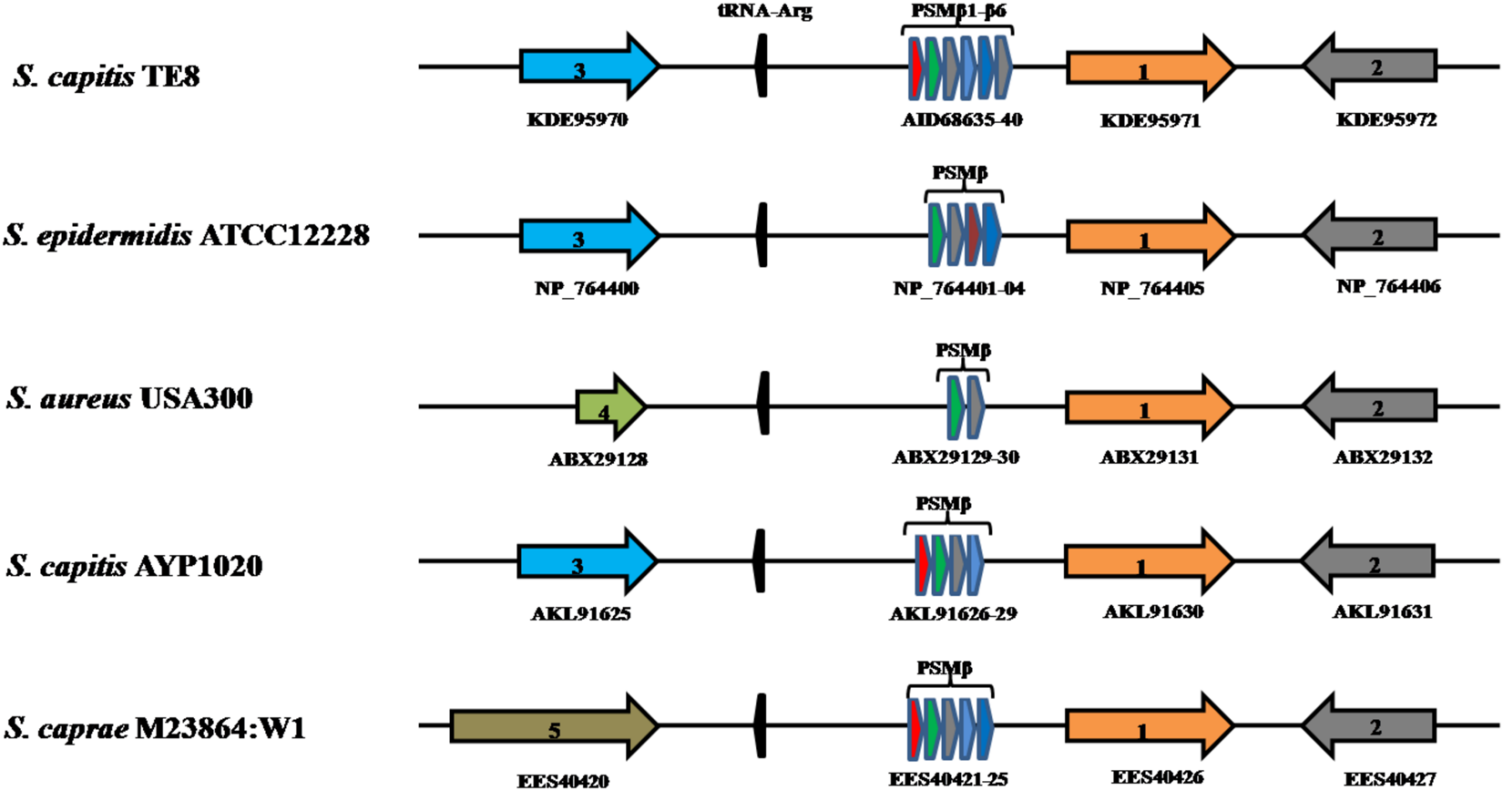
Gene organization of putative PSMβ type peptides of *Staphylococcus capitis* TE8. Genetic loci coding for PSMβ peptides in the genome of various *Staphylococcus* spp. 1- HAD-like hydrolases superfamily, 2- NAT-SF superfamily, 3- phosphoesterase, 4- hypothetical protein, 5- MFS transporter.

A gene cluster encoding six PSMβ-type peptides (PSMβ1-β6) was identified in *S. capitis* TE8 using RAST server. The number of PSMβ peptides encoded in the genomes of closely related staphylococci usually varies from two to six (Fig. 7). All the six predicted PSMβ peptides are closely related to each other with identity ranging from 55% to 93% (Supplemental Table S3) and contain the conserved domain of staph_haemo superfamilies.

RAST server identified another gene encoding a hypothetical peptide, HTP2388 (AIK23238) with a conserved domain of staph_haemo superfamilies. HTP2388 shows 27% identity to PSMβ1 and low similarity to other identified PSMβ peptides encoded in the *S. capitis* TE8 genome. A BLAST search for similar peptides in other bacterial genomes identified several hits suggesting that the peptide is conserved across several staphylococci genomes. The sequence of HTP2388 was found to have 100% sequence identity to several other peptides, namely, hypothetical protein (YP_006939115) of *S. epidermidis*, a hemolysin-like peptide (WP_002436888) of *S. capitis*, a hypothetical protein (EEE48298) of *S. capitis* SK14 and to another hypothetical protein (EES40760) of *S. caprae*.

**Figure 8:**
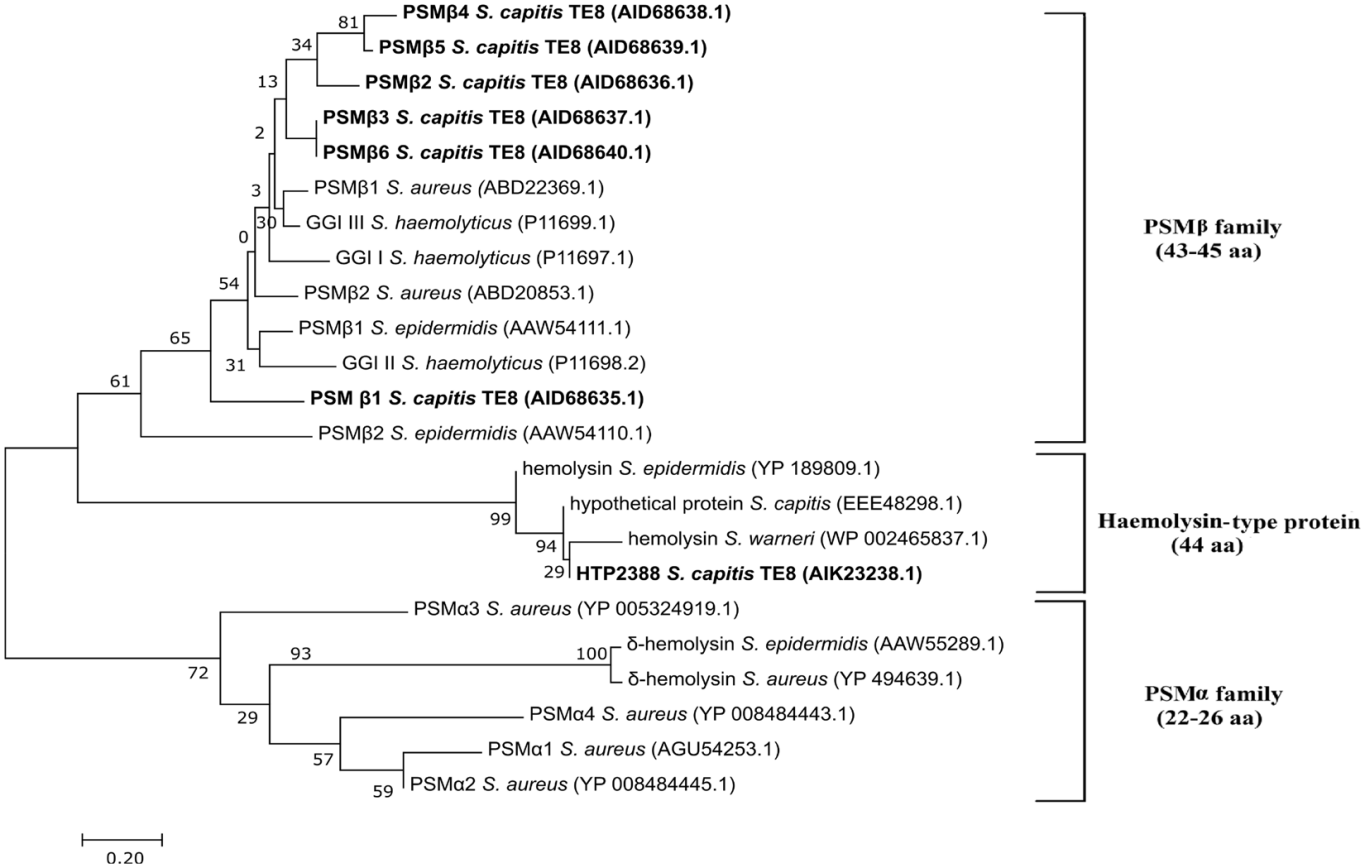
Phylogenetic analysis of antibacterial peptides from *Staphylococcus capitis* TE8 along with other peptides of PSM family. Neighbor-joining phylogenetic tree of PSMβ peptides (PSMβ1-β6) and hypothetical peptide HTP2388 of *S. capitis* TE8 and PSM of other *Staphylococcus* spp. Evolutionary analyses were performed using MEGA7.

The conserved staph_haemo domain found in PSMβ1-β6 and in HTP2388 is present in several small staphylococcal proteins, such as SLUSH proteins, haemolysin, gonococcal growth inhibitors and PSMβ peptides. Phylogenetic analysis of PSMβ and HTP2338 showed the clustering of predicted PSMβ peptides of *S. capitis* TE8 with the previously reported PSMβ peptides of various *Staphylococcus* species whereas the peptide HTP2388 clusters with other putative hemolysin-like proteins of staphylococci, confirming it to be a member of a separate family (Fig. 8). All the predicted PSMβ-type peptides of *S. capitis* TE8 are phylogenetically closer to PSMβ of *S. aureus* and gonococcal growth inhibitors. Gonococcal growth inhibitors are 44 amino acid peptides which are recently included in PSMβ families on the basis of size and are known to have inhibitory activity against *Neisseria gonorrhoeae* ^19^. PSMs have been shown to be involved in Staphylococcal pathogenesis and virulence and play a major role in survival of staphylococci on epithelial surfaces^19^. PSMβ peptides are devoid of signal peptides and are known to be secreted by PMT transporter in *S. aureus* ^20^. A putative PMT transporter was also identified in the genome of *S. capitis* TE8 consisting of four genes (*pmtA, pmtB, pmtC* and *pmtD*), which may be involved in secretion of PSMβ peptides.

### Synthetic PSMβ1-6 and HTP2388 peptides exhibit antimicrobial activity

**Figure 9:**
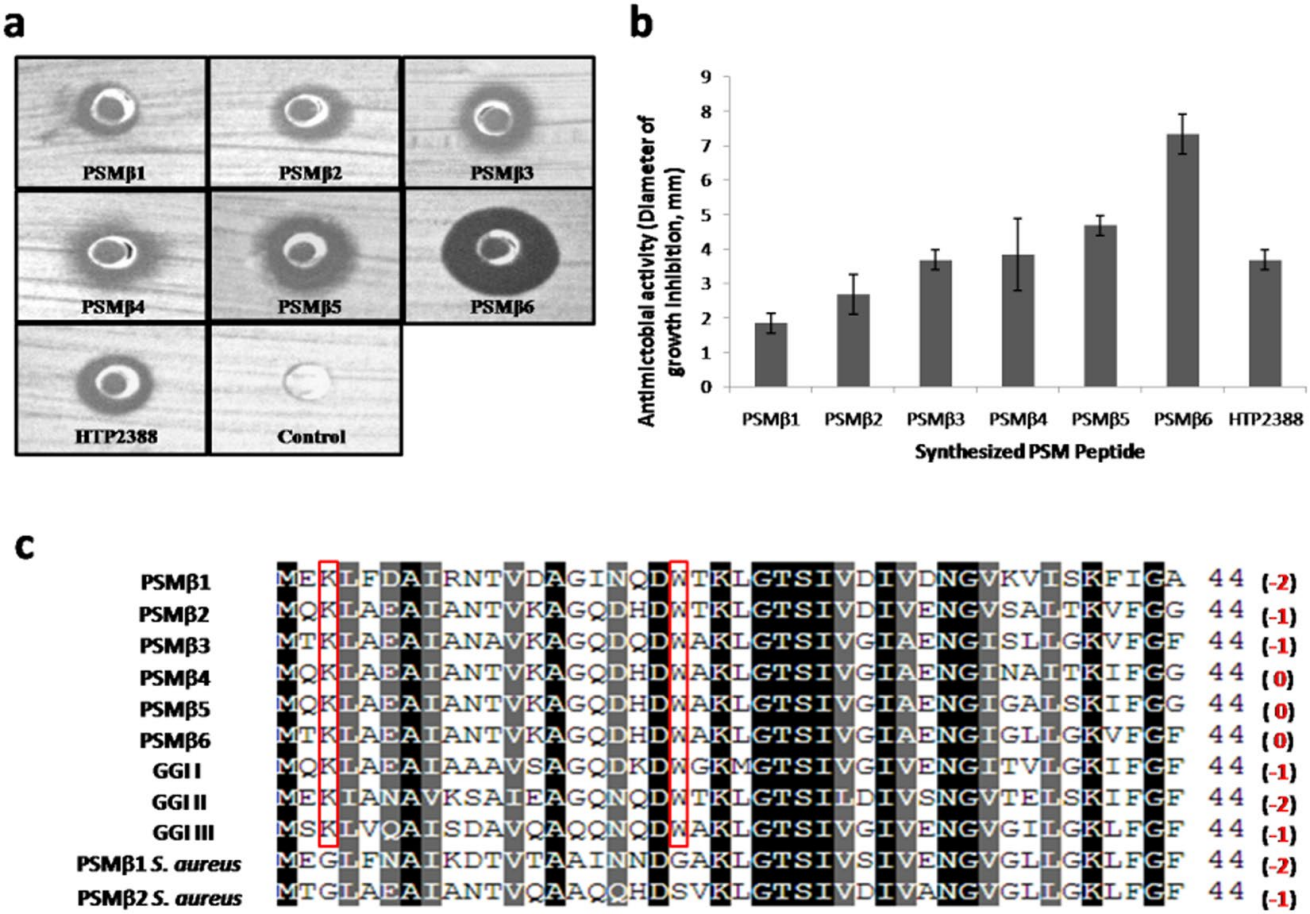
Antibacterial activity of synthesized peptides PSMβ1-β6 and HTP2388. (**a**) and (**b**) Antibacterial activity of synthesized peptides. Peptides were dissolved in DMSO: water (1:1) and agar well diffusion assay were done against *Micrococcus luteus* test plate. Test plates were grown overnight and zones of inhibition were measured. (**c**) Sequence alignment of PSMβ1-β6 of *Staphylococcus capitis* TE8 along with gonococcal growth inhibitors from *Staphylococcus haemolyticus* [GGI I (P11697.1), GGI II (P11698.1), GGI III (P11699.1)], PSMβ1 (ABD22369.1) and PSMβ2 (ABD20853.1) of *Staphylococcus aureus* USA300_FPR3757. Net charge on peptides was written in the parentheses.

The six putative PSMβ peptides and peptide HTP2388 from *S. capitis* TE8 were synthesized by GL Biochem (Shanghai) Ltd. and tested for possible antimicrobial activity. All the seven synthetic peptides were found to have antibacterial activity against *M. luteus* with the highest activity in PSMβ6 and least in PSMβ1 (Fig. 9a and b). Further analysis suggested that net charge on the peptides may play a role in the observed antimicrobial activity as also reported earlier^21^. PSMβ4, PSMβ5, PSMβ6 having neutral net charge showed higher antibacterial activity in comparison to the negatively charged peptides (PSMβ1, PSMβ2, PSMβ3). PSMβ1/β2 of *S. aureus* with net negative charge are also reported to have no significant antibacterial activity^22^. Sequence comparison of PSMβ1-6 of *S. capitis* TE8 with PSMβ1/β2 of *S. aureus* and gonococcal growth inhibitor peptide sequences of *S. aureus* indicated the importance of conserved residues to maintain the amphipathic nature of the peptides and in imparting antimicrobial activity (Fig. 9c and Supplemental Fig. S3). A lysine at 3^rd^ and a tryptophan at 20^th^ residue position were found to be conserved across gonococcal growth inhibitors as well as in PSMβ peptides of *S. capitis* TE8, which have been demonstrated to have antibacterial activity. These residues were found to be replaced by glycine in both PSMβ1/β2 of *S. aureus*, which do not possess antibacterial activity^22^. It is tempting to associate the role of lysine at 3^rd^ and/or tryptophan at 20^th^ position in providing antibacterial activity of PSMβ family peptides but would require further validation on a larger set of PSMβ family peptides from different organisms.

### Genomic insights into adaptation of *Staphylococcus capitis* TE8 on skin

*S. capitis* TE8 was isolated from the human skin and expected to harbour genes required for its invasion, adhesion and virulence. Detailed sequence analysis of *S. capitis* TE8 genome identified several putative proteins required for adherence, invasion, virulence, biofilm formation, osmotolerance and acid tolerance (Supplemental Table S2). This gamut of genes is expected to play important role in adherence and colonization of strain TE8 on skin. Strain TE8 possess 14 adhesins including 7 cell wall anchored proteins with LPXTG motif, a virulence associated cell wall anchored protein, 4 bifunctional autolysins, a SdrD-like adhesion and an elastin binding protein. *S. capitis* TE8 genome also contains genes for intercellular adhesions (*icaADBC)* and the regulatory gene *icaR*. The genes in *ica* locus are found to be important for biofilm formation in *S. aureus* and *S. epidermidis* ^23^. Biofilm formation in *S. epidermidis* is known as a beneficial mechanism in order to get resistance against various antibiotics and protection from host defence system^24^. Development of biofilm formation begins with primary attachment of bacteria to surface and human matrix proteins. Microbial surface components recognizing adhesive matrix molecules (MSCRAMMs) are required for initial attachment of *S. aureus* and *S. epidermidis* to human matrix proteins^25, 26^. A bifunctional adhesion and autolysin (AtlE) surface protein also has been shown to play important roles in adhesion to abiotic surface^27^. The 14 putative adhesins (including 4 bifunctional autolysins) along with intracellular adhesins may be responsible for initial attachment and colonization of *S. capitis* TE8 on human skin.

Apart from possessing double the number of adhesins, *S. capitis* strains also possess genes for aureusimine (Supplemental Table S4) in comparison to *S. caprae* and *S. epidermidis*. Genes for cAMP signalling, LysR family regulatory proteins, Grams positive competence and late competence genes, mannitol utilization, twin arginine translocation system and restriction modification systems were present in *S. capitis* TE8, *S. capitis* AYP1020 and *S. caprae* but were absent in *S. epidermidis*, while genes for lactose and galactose uptake and utilization, butanol biosynthesis and mixed acid fermentation genes were present only in *S. epidermidis*. Mannitol utilization is shown to be important for protection of *S. aureus* from human skin antimicrobial fatty acids^28^; also disruption of twin-arginine translocation pathway in *S. aureus* resulted in significantly reduced virulence in mouse kidney abscess model^29^. A putative LysR family transcription factor and a putative cyclic di-GMP synthetase were shown to regulate hemolysin expression in *S. aureus* ^30^. Presence of higher number of adhesins, auresusimine biosynthesis, mannitol utilization, LysR family regulatory protein may indicate towards higher adaptability and virulence of *S capitis* TE8 and *S. capitis* AYP1020 in comparison to *S. epidermidis*.

Skin is the first physical barrier to the environment. Skin microbiome is hence likely to be exposed to various stress factors and the members of skin microbiome expected to possess mechanisms of stress tolerance for survival on the skin surface. Strain TE8 genome has 16 genes for osmoadaptation, which include 2 putative OpuC-type osmolyte uptake systems, 2 putative betaine uptake systems, genes for choline uptake and conversion to glycine-betaine and 2 putative low affinity proline transporters. To overcome the oxidative stress, *S. capitis* TE8 possess 27 genes encoding for oxidative stress tolerance including genes for superoxide dismutase (SOD), catalase, ferroxidase and OsmC. The strain contains a eukaryotic-type low affinity urea transporter and all components for a functional urease. Along with this, the strain also possesses arginine and ornithine degradation pathway, which are expected to help in adaptation to acidic skin environment. The presence of a large gamut of osmotic/salinity stress, oxidative and acid stress protection mechanisms in *S. capitis* TE8 may be a reflection on adaptation of this strain to human skin.

## Conclusion

We have isolated a skin resident bacterium with the ability to produce a repertoire of antimicrobial peptides with activity against Gram positive strains including *S. aureus*. Comparative genomics revealed features which might be responsible for adaptation and colonization of this strain on human skin.

## Methods

### Sample isolation and antimicrobial activity assay

For isolation of skin microbiota, consent from human subjects and permission of Institutional Ethical Committee of CSIR-Institute of Genomics and Integrative Biology, New Delhi 110020, India was taken before the sampling. Guidelines of the Institutional Ethical Committee were followed for the work. *S. capitis* TE8 was isolated from human skin of healthy volunteer using a sterile cotton swab, pre-soaked in a sterile saline solution containing 0.15 M NaCl and 0.1% Tween-20 and grown on LB agar at 37 °C. It was screened for antimicrobial activity using agar overlay method. The antagonistic property of the isolate was checked against *B. subtilis, C. glabrata*, *E. coli, M. luteus, P. aeruginosa* and *S. aureus*. Briefly, the strain was first grown on LB agar plates at 37 °C for 48 h and then an overlay of LB top agar (0.7%) with the above mentioned test strains, pre-grown to A_600_ of 0.4–0.5, was carried out on the plates. The plates were then incubated at 37 °C overnight, followed by the detection of the antimicrobial activity based on the zone of inhibition.

### Bacterial identification and phylogenetic analysis

Isolate TE8 exhibiting antimicrobial activity against the test strains was further grown in Luria Bertani (LB) medium (pH 7.2) in a shaking incubator at 37 °C with 200 rpm for identification and further analysis. This isolate was identified by rrs (16S rRNA) gene sequence analysis. Briefly, *rrs* gene was amplified from the genomic DNA using universal primers, 27 F (AGAGTTTGATCMTGGCTCAG) and 1492 R (TACGGYTACCTTGTTACGACTT). The amplified product was sequenced and the obtained sequence was used to identify the bacterium and its closely related members using BLAST and EzTaxon^31^. The sequence of the amplified region of 16S rRNA was submitted to NCBI with an accession number of KJ420579.

Phylogenetic analysis of the 16S rRNA gene sequence of *S. capitis* TE8 with other *rrs* gene sequences obtained from BLAST analysis was carried out by the Maximum Likelihood method based on the Le Gascuel, 2008 model in MEGA v.7.0^32^ and the gaps were treated using partial deletion method.

### Extraction of antimicrobial agents

Extraction of agents exhibiting antimicrobial activity was done using 1-butanol extraction method^22^. In brief, isolate TE8 was grown in 100 ml of LB medium at 37 °C for 24 h. The cells were then harvested by centrifugation at 8228 × g for 10 min and the supernatant collected. The supernatant was mixed with 30 ml of 1-butanol (3:1 ratio) and extraction of secreted antimicrobial compounds was carried out by shaking the mixed solutions at room temperature for 2 h. After brief centrifugation 1-butanol phase was collected and dried by vacuum centrifugation. Dried sample was dissolved in 200 μl of sterile water and 50 μl was checked again for antagonistic properties against *B. subtilis*, *M. luteus* and *S. aureus*.

### Effect of Proteinase K enzyme on antibacterial activity

To confirm whether the antimicrobial activity is due to a proteinaceous agent or other secondary metabolites, the extract was treated with Proteinase K (Proteinase K:1-butanol extract:: 1:10) at 37 °C for 1 h. The proteinase K treated extract was checked for antibacterial activity against *M. luteus* using agar well diffusion assay. After overnight incubation, zone of inhibition was measured to monitor the residual antibacterial activity.

### Genome sequencing and analysis

To extract the genomic DNA of isolate TE8, the culture was grown in 5 ml LB broth for 14–16 h. The cells were harvested by centrifugation at 9000 g for 10 min and the genomic DNA was isolated using Microbial DNA purification kit (Mo Bio Laboratories Inc) according the manufacturer’s protocol.

The genome sequencing of isolate TE8 was carried out with a combined strategy using whole genome shotgun (Roche GS-Junior) and paired-end pyrosequencing (3 kb) (Roche FLX). *De novo* assembly of the raw reads was done by Newbler v2.9 assembler software. Assembled contigs were used for genome annotation by NCBI Prokaryotic Genome Annotation pipeline (PGAP) and Rapid Annotation using Subsystems Technology (RAST) server^33^, which use tRNAscan-SE and GLIMMER2 for prediction of tRNA and protein encoding genes, respectively. The automated annotation was followed by manual curation/inspection. The genomic islands were predicted using IslandViewer4.^34^ Secondary metabolites were predicted by Antibiotics & Secondary Metabolite Analysis Shell (antiSMASH)^35^ and bacteriocin were identified by BAGEL3^36^.

The genome and protein sequences of *S. capitis* subsp*. capitis* AYP1020^37^ (GCA_001028645.1), *S. caprae* M23864:W1 (GCA_000160215.1) and *S. epidermidis* ATCC 12228^38^ (GCA_000007645.1) were downloaded from NCBI Repository for in-silico DNA to DNA hybridization (DDH) and comparative genomics analysis. Accession numbers and general features of the selected genomes are provided in Supplemental Table S5. In-silico prediction of DDH value was calculated by using Genome-to-Genome Distance Calculator tool 2.1^39^. Grouping of the proteins into orthologs based on their sequence similarity was performed by OrthoMCL^40^. OrthoMCL uses the reciprocal best BLAST hit approach and makes an adjustment for species distance (normalization) to distinguish orthologs from in-paralogs and the resulting groups are generated from the normalized BLAST scores between proteins using Markov Clustering. The set of orthologous genes for each strain determined by orthoMCL were presented in the form of a Venn diagram using Venny 2.1 (http://bioinfogp.cnb.csic.es/tools/venny/index.html).

### Synthesis and activity of antimicrobial peptides

The predicted six PSMβ peptides (PSMβ1-β6) and the hypothetical peptide HTP2388 were commercially synthesised by GL Biochem (Shanghai) Ltd. at purity greater than 95%. Synthesized peptides were dissolved to a concentration of 10 mg/ml in DMSO: water (1:1) and 20 µl of this was checked for antibacterial activity against *M. luteus*. DMSO:water (1:1) without any peptide was used as a control.

### Nucleotide sequence accession numbers

The genome sequence of *S. capitis* TE8 has been deposited at GenBank under the accession no. JMGB00000000. The gene clusters of epidermicin, gallidermin, six phenol soluble modulin-like peptides and peptide HTP2388 were deposited in GenBank under accession #KJ702950, KJ728983, KJ702949 and KJ867527, respectively.

### Ethical statement

The Ethical Committee of the CSIR-Institute of Genomics and Integrative Biology has approved the work and the subjects gave informed consent to the work.

## Electronic supplementary material

Supplementary information
