# Supplementary material for: Genome Analysis of Staphylococcus capitis TE8 Reveals Repertoire of Antimicrobial Peptides and Adaptation Strategies for Growth on Human Skin
Source: Sci Rep. 2017 Sep 5;7:10447. doi: 10.1038/s41598-017-11020-7 (PMC5585272; doi:10.1038/s41598-017-11020-7)
Supplement: Supplementary file 1 — Supplementary information [file 41598_2017_11020_MOESM1_ESM.pdf]

**Supplementary Data**

**Title: Genome Analysis of *Staphylococcus capitis* TE8 Reveals Repertoire of Antimicrobial Peptides and Adaptation Strategies for Growth on Human Skin.**

**Short title:** Repertoire of Antimicrobial Peptides and Adaptation Strategies of *S. capitis* TE8.

**Rohit Kumar[1,#], Pramod Kumar Jangir[1,$], Jhumki Das[1], Bhupesh Taneja[1,2*], Rakesh Sharma[1,2*]**

[1]CSIR-Institute of Genomics and Integrative Biology, Council of Scientific and Industrial Research (CSIR), New Delhi, India

[2]Academy of Scientific and Innovative Research (AcSIR), New Delhi, India

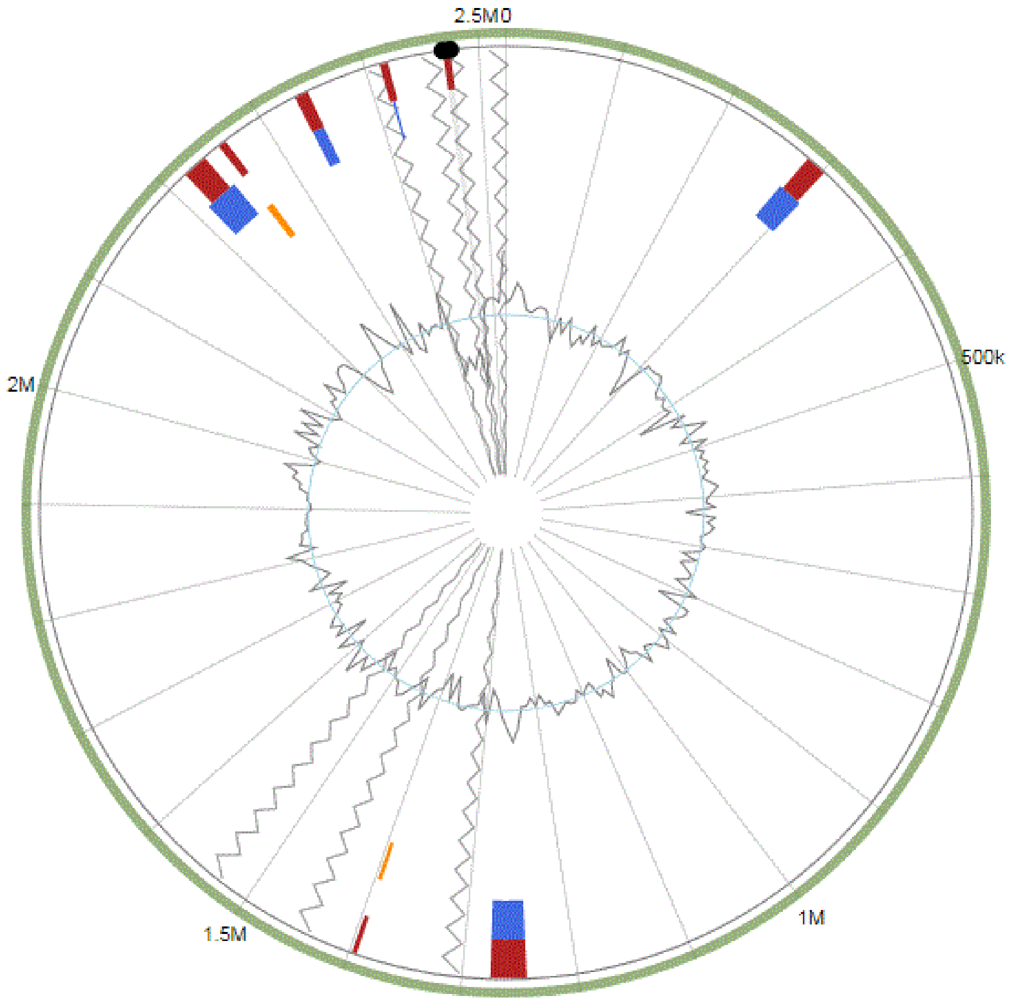


**Figure S1. Genomic island (GI) analysis of *Staphylococcus capitis* TE8 was conducted using IslandViewer 4. Alignment of the genome was done against *Staphylococcus capitis* AYP1020 genome. Epidermicin was located in the marked GI.**

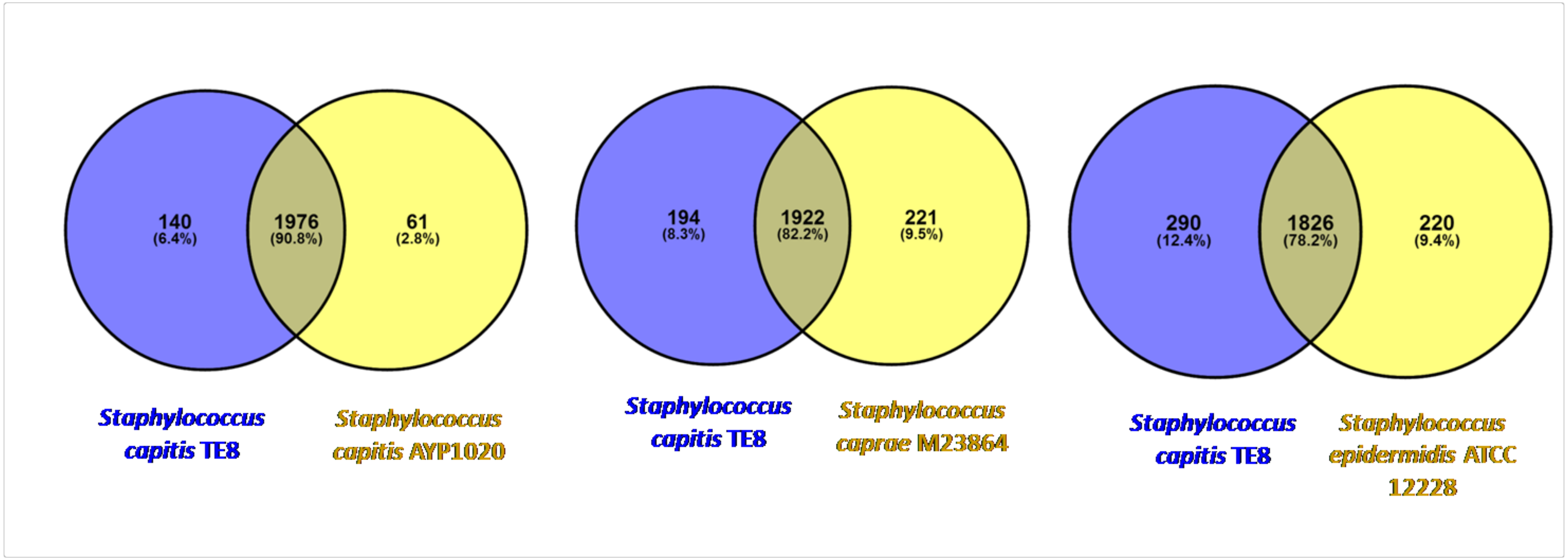


**Figure S2. Venn diagram showing the core and strain specific proteins in comparison with *Staphylococcus capitis* TE8 with strains of *Staphylococcus* spp. OrthoMCL v2.0.9 was used to generate clusters of orthologous proteins that are indicated by overlapping regions.**

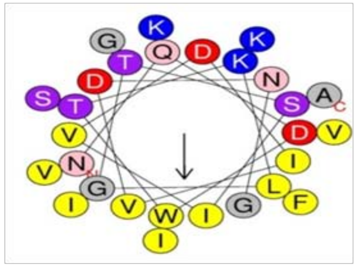

**PSMβ1(-2)**

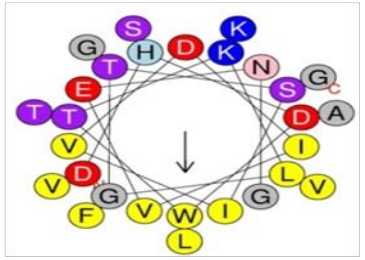

**PSMβ2(-1)**

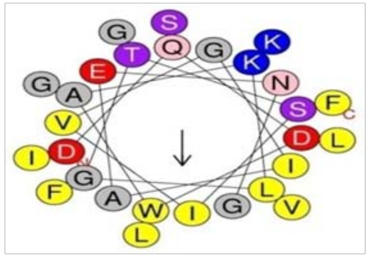

**PSMβ3( -1)**

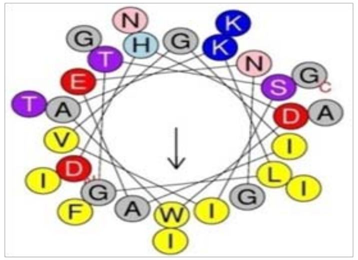

**PSMβ4(0)**

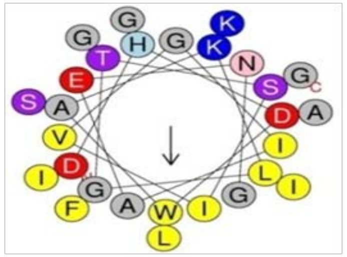

**PSMβ5( 0)**

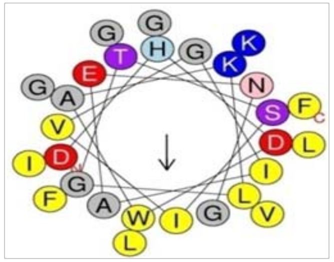

**PSMβ6( 0)**

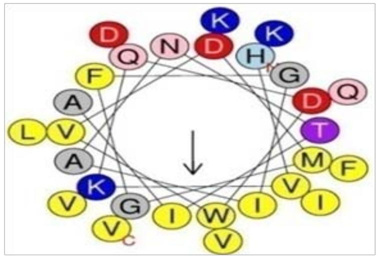

**HTP2388( 0)**

**Figure S3. Predicted helix plot of identified PSMβ peptides and hypothetical protein of strain TE8. α-helical wheel arrangement (17-44 amino acid) of antibacterial peptides PSMβ1-β6 and HTP2388 showing amphipathy (http://heliquest.ipmc.cnrs.fr)**

**Table S1. Genomic Islands detected in *Staphylococcus capitis* TE8 genome.**

| Island start | Island end | Length | Method | Gene start | Gene end | Strand | Product |
|---|---|---|---|---|---|---|---|
| 283532 | 302105 | 18573 | IslandPath-DIMOB | 283532 | 284176 | 1 | Hypothetical SAV0794 homolog in superantigen-encoding pathogenicity islands SaPI |
| 283532 | 302105 | 18573 | IslandPath-DIMOB | 284544 | 284876 | 1 | Hypothetical SAV0795 homolog in superantigen-encoding pathogenicity islands SaPI |
| 283532 | 302105 | 18573 | IslandPath-DIMOB | 284886 | 285638 | 1 | Hypothetical SAV0798 homolog in superantigen-encoding pathogenicity islands SaPI |
| 283532 | 302105 | 18573 | IslandPath-DIMOB | 285653 | 286240 | 1 | Hypothetical SAV0796 homolog in superantigen-encoding pathogenicity islands SaPI |
| 283532 | 302105 | 18573 | IslandPath-DIMOB | 286240 | 286449 | 1 | FIG01110123: hypothetical protein |
| 283532 | 302105 | 18573 | IslandPath-DIMOB | 286504 | 286941 | 1 | Hypothetical SAV0798 homolog in superantigen-encoding pathogenicity islands SaPI |
| 283532 | 302105 | 18573 | IslandPath-DIMOB | 286938 | 287279 | 1 | Hypothetical SAV0799 homolog in superantigen-encoding pathogenicity islands SaPI |
| 283532 | 302105 | 18573 | IslandPath-DIMOB | 287281 | 287853 | 1 | Putative terminase, superantigen-encoding pathogenicity islands SaPI |
| 283532 | 302105 | 18573 | IslandPath-DIMOB | 287961 | 288128 | 1 | hypothetical protein |
| 283532 | 302105 | 18573 | IslandPath-DIMOB | 288407 | 288967 | 1 | hypothetical protein |
| 283532 | 302105 | 18573 | IslandPath-DIMOB | 288964 | 289407 | 1 | hypothetical protein |
| 283532 | 302105 | 18573 | IslandPath-DIMOB | 290245 | 290361 | 1 | hypothetical protein |
| 283532 | 302105 | 18573 | IslandPath-DIMOB | 290569 | 290793 | 1 | hypothetical protein |
| 283532 | 302105 | 18573 | IslandPath-DIMOB | 291579 | 292040 | -1 | hypothetical protein |

| | | | | | | | |
|---|---|---|---|---|---|---|---|
| 283532 | 302105 | 18573 | IslandPath-DIMOB | 292132 | 292290 | -1 | hypothetical protein |
| 283532 | 302105 | 18573 | IslandPath-DIMOB | 292401 | 292832 | 1 | acetyltransferase, GNAT family |
| 283532 | 302105 | 18573 | IslandPath-DIMOB | 292956 | 293528 | -1 | hypothetical protein |
| 283532 | 302105 | 18573 | IslandPath-DIMOB | 294535 | 294735 | 1 | Cold shock protein CspC |
| 283532 | 302105 | 18573 | IslandPath-DIMOB | 294858 | 295592 | 1 | hypothetical protein |
| 283532 | 302105 | 18573 | IslandPath-DIMOB | 295901 | 296125 | -1 | FIG01108328: hypothetical protein |
| 283532 | 302105 | 18573 | IslandPath-DIMOB | 296149 | 296433 | -1 | FIG01108459: hypothetical protein |
| 283532 | 302105 | 18573 | IslandPath-DIMOB | 296553 | 297299 | -1 | phage-related protein |
| 283532 | 302105 | 18573 | IslandPath-DIMOB | 297480 | 298085 | 1 | Acetyltransferase( EC:2.3.1.- ) |
| 283532 | 302105 | 18573 | IslandPath-DIMOB | 298219 | 298452 | 1 | FIG01109356: hypothetical protein |
| 283532 | 302105 | 18573 | IslandPath-DIMOB | 298514 | 299098 | -1 | FIG01107922: hypothetical protein |
| 283532 | 302105 | 18573 | IslandPath-DIMOB | 299291 | 299476 | -1 | FIG01107975: hypothetical protein |
| 283532 | 302105 | 18573 | IslandPath-DIMOB | 300205 | 300408 | 1 | FIG01108340: hypothetical protein |
| 283532 | 302105 | 18573 | IslandPath-DIMOB | 300400 | 300636 | -1 | FIG01108334: hypothetical protein |
| 283532 | 302105 | 18573 | IslandPath-DIMOB | 300848 | 301015 | 1 | Hypothetical, related to broad specificity phosphatases COG0406 |
| 283532 | 302105 | 18573 | IslandPath-DIMOB | 301055 | 301432 | 1 | Hypothetical, related to broad specificity phosphatases COG0406 |

| | | | | | | | |
|---|---|---|---|---|---|---|---|
| 283532 | 302105 | 18573 | IslandPath-DIMOB | 301488 | 302105 | -1 | Transporter, LysE family |
| 1243992 | 1275497 | 31505 | IslandPath-DIMOB | 1243992 | 1244390 | -1 | FIG01108908: hypothetical protein |
| 1243992 | 1275497 | 31505 | IslandPath-DIMOB | 1244371 | 1244793 | -1 | FIG01109699: hypothetical protein |
| 1243992 | 1275497 | 31505 | IslandPath-DIMOB | 1244807 | 1245994 | -1 | FIG01108548: hypothetical protein |
| 1243992 | 1275497 | 31505 | IslandPath-DIMOB | 1246007 | 1247893 | -1 | Putative major teichoic acid biosynthesis protein C |
| 1243992 | 1275497 | 31505 | IslandPath-DIMOB | 1247896 | 1249356 | -1 | Phage minor structural protein |
| 1243992 | 1275497 | 31505 | IslandPath-DIMOB | 1249368 | 1250324 | -1 | Phage protein |
| 1243992 | 1275497 | 31505 | IslandPath-DIMOB | 1250336 | 1254244 | -1 | Phage tail length tape-measure protein |
| 1243992 | 1275497 | 31505 | IslandPath-DIMOB | 1254259 | 1254561 | -1 | Phage protein |
| 1243992 | 1275497 | 31505 | IslandPath-DIMOB | 1254645 | 1255004 | -1 | Phage protein |
| 1243992 | 1275497 | 31505 | IslandPath-DIMOB | 1255069 | 1255626 | -1 | Phage tail protein |
| 1243992 | 1275497 | 31505 | IslandPath-DIMOB | 1255669 | 1256058 | -1 | Phage protein |
| 1243992 | 1275497 | 31505 | IslandPath-DIMOB | 1256058 | 1256420 | -1 | Phage protein |
| 1243992 | 1275497 | 31505 | IslandPath-DIMOB | 1256420 | 1256722 | -1 | Phage protein |
| 1243992 | 1275497 | 31505 | IslandPath-DIMOB | 1256719 | 1257048 | -1 | phi 11 orf36 homolog [SA bacteriophages 11, Mu50B] |
| 1243992 | 1275497 | 31505 | IslandPath-DIMOB | 1257050 | 1257319 | -1 | hypothetical protein |

| | | | | | | | |
|---|---|---|---|---|---|---|---|
| 1243992 | 1275497 | 31505 | IslandPath-DIMOB | 1257340 | 1258269 | -1 | Phage major capsid protein |
| 1243992 | 1275497 | 31505 | IslandPath-DIMOB | 1258286 | 1258897 | -1 | hypothetical protein |
| 1243992 | 1275497 | 31505 | IslandPath-DIMOB | 1259067 | 1259210 | -1 | hypothetical protein |
| 1243992 | 1275497 | 31505 | IslandPath-DIMOB | 1259203 | 1259748 | -1 | hypothetical protein |
| 1243992 | 1275497 | 31505 | IslandPath-DIMOB | 1259768 | 1260718 | -1 | FIG01109806: hypothetical protein |
| 1243992 | 1275497 | 31505 | IslandPath-DIMOB | 1260725 | 1262251 | -1 | Phage portal |
| 1243992 | 1275497 | 31505 | IslandPath-DIMOB | 1262264 | 1263550 | -1 | Phage terminase, large subunit |
| 1243992 | 1275497 | 31505 | IslandPath-DIMOB | 1263553 | 1263939 | -1 | Phage terminase, small subunit |
| 1243992 | 1275497 | 31505 | IslandPath-DIMOB | 1264189 | 1264605 | -1 | phage transcriptional regulator, RinA family |
| 1243992 | 1275497 | 31505 | IslandPath-DIMOB | 1264676 | 1264846 | -1 | FIG01109198: hypothetical protein |
| 1243992 | 1275497 | 31505 | IslandPath-DIMOB | 1264852 | 1264992 | -1 | hypothetical protein within prophage |
| 1243992 | 1275497 | 31505 | IslandPath-DIMOB | 1264997 | 1265173 | -1 | hypothetical protein |
| 1243992 | 1275497 | 31505 | IslandPath-DIMOB | 1265210 | 1265635 | -1 | Deoxyuridine 5'-triphosphate nucleotidohydrolase (EC 3.6.1.23) |
| 1243992 | 1275497 | 31505 | IslandPath-DIMOB | 1265653 | 1266231 | -1 | hypothetical protein |
| 1243992 | 1275497 | 31505 | IslandPath-DIMOB | 1266224 | 1266565 | -1 | hypothetical protein |
| 1243992 | 1275497 | 31505 | IslandPath-DIMOB | 1266571 | 1266753 | -1 | hypothetical protein |

| | | | | | | | |
|---|---|---|---|---|---|---|---|
| 1243992 | 1275497 | 31505 | IslandPath-DIMOB | 1266754 | 1267101 | -1 | phiPVL ORF050-like protein |
| 1243992 | 1275497 | 31505 | IslandPath-DIMOB | 1267102 | 1267293 | -1 | Phage protein |
| 1243992 | 1275497 | 31505 | IslandPath-DIMOB | 1267295 | 1267474 | -1 | conserved phage protein |
| 1243992 | 1275497 | 31505 | IslandPath-DIMOB | 1267477 | 1267632 | -1 | hypothetical protein |
| 1243992 | 1275497 | 31505 | IslandPath-DIMOB | 1267626 | 1268396 | -1 | DNA replication protein DnaC |
| 1243992 | 1275497 | 31505 | IslandPath-DIMOB | 1268407 | 1269192 | -1 | Phage replication initiation protein |
| 1243992 | 1275497 | 31505 | IslandPath-DIMOB | 1269179 | 1269940 | -1 | HNH endonuclease |
| 1243992 | 1275497 | 31505 | IslandPath-DIMOB | 1269937 | 1270608 | -1 | Hypothetical protein, PV83 orf19 homolog [SA bacteriophages 11, Mu50B] |
| 1243992 | 1275497 | 31505 | IslandPath-DIMOB | 1270620 | 1271165 | -1 | Single stranded DNA-binding protein, phage-associated |
| 1243992 | 1275497 | 31505 | IslandPath-DIMOB | 1271197 | 1271982 | -1 | Hypothetical protein, phi-ETA orf17 homolog [SA bacteriophages 11, Mu50B] |
| 1243992 | 1275497 | 31505 | IslandPath-DIMOB | 1271983 | 1272468 | -1 | Phage protein |
| 1243992 | 1275497 | 31505 | IslandPath-DIMOB | 1272461 | 1272715 | -1 | hypothetical protein |
| 1243992 | 1275497 | 31505 | IslandPath-DIMOB | 1272779 | 1272955 | -1 | hypothetical protein |
| 1243992 | 1275497 | 31505 | IslandPath-DIMOB | 1273111 | 1273344 | 1 | Phage protein |
| 1243992 | 1275497 | 31505 | IslandPath-DIMOB | 1273328 | 1273492 | -1 | hypothetical protein |
| 1243992 | 1275497 | 31505 | IslandPath-DIMOB | 1273506 | 1273715 | -1 | Phage protein |

| | | | | | | | |
|---|---|---|---|---|---|---|---|
| 1243992 | 1275497 | 31505 | IslandPath-DIMOB | 1273770 | 1273952 | 1 | hypothetical protein |
| 1243992 | 1275497 | 31505 | IslandPath-DIMOB | 1273933 | 1274088 | -1 | hypothetical protein |
| 1243992 | 1275497 | 31505 | IslandPath-DIMOB | 1274176 | 1274514 | 1 | Phage protein |
| 1243992 | 1275497 | 31505 | IslandPath-DIMOB | 1274500 | 1274733 | -1 | hypothetical protein |
| 1243992 | 1275497 | 31505 | IslandPath-DIMOB | 1275165 | 1275497 | 1 | Phage protein |
| 1393102 | 1397347 | 4245 | SIGI-HMM | 1393102 | 1393746 | -1 | hypothetical protein |
| 1393102 | 1397347 | 4245 | SIGI-HMM | 1394200 | 1394934 | -1 | uncharacterized phage-associated protein |
| 1393102 | 1397347 | 4245 | SIGI-HMM | 1394948 | 1395598 | -1 | Phage protein |
| 1393102 | 1397347 | 4245 | SIGI-HMM | 1396662 | 1397045 | -1 | hypothetical protein |
| 1393102 | 1397347 | 4245 | SIGI-HMM | 1397159 | 1397347 | -1 | hypothetical protein |
| 2218766 | 2241087 | 22321 | IslandPath-DIMOB | 2218766 | 2220097 | -1 | hypothetical protein |
| 2218766 | 2241087 | 22321 | IslandPath-DIMOB | 2220084 | 2222090 | -1 | Endonuclease |
| 2218766 | 2241087 | 22321 | IslandPath-DIMOB | 2222345 | 2223157 | 1 | hypothetical protein |
| 2218766 | 2241087 | 22321 | IslandPath-DIMOB | 2223391 | 2223843 | -1 | Type I restriction-modification system, specificity subunit S (EC 3.1.21.3) |
| 2218766 | 2241087 | 22321 | IslandPath-DIMOB | 2224145 | 2224600 | -1 | Type I restriction-modification system, specificity subunit S (EC 3.1.21.3) |
| 2218766 | 2241087 | 22321 | IslandPath-DIMOB | 2224593 | 2226149 | -1 | Type I restriction-modification system, DNA-methyltransferase subunit M (EC 2.1.1.72) |
| 2218766 | 2241087 | 22321 | IslandPath-DIMOB | 2226360 | 2227256 | -1 | hypothetical protein |
| 2218766 | 2241087 | 22321 | IslandPath-DIMOB | 2227300 | 2228856 | -1 | Sll1503 protein |

| | | | | | | | |
|---|---|---|---|---|---|---|---|
| 2218766 | 2241087 | 22321 | IslandPath-DIMOB | 2229131 | 2229259 | -1 | FIG01108249: hypothetical protein |
| 2218766 | 2241087 | 22321 | IslandPath-DIMOB | 2229273 | 2230157 | -1 | Transporter |
| 2218766 | 2241087 | 22321 | IslandPath-DIMOB | 2230245 | 2230565 | -1 | Arsenical resistance operon repressor |
| 2218766 | 2241087 | 22321 | IslandPath-DIMOB | 2230611 | 2230805 | -1 | Similarity |
| 2218766 | 2241087 | 22321 | IslandPath-DIMOB | 2231088 | 2231435 | 1 | Arsenical resistance operon trans-acting repressor ArsD |
| 2218766 | 2241087 | 22321 | IslandPath-DIMOB | 2231416 | 2233143 | 1 | Arsenical pump-driving ATPase (EC 3.6.3.16) |
| 2218766 | 2241087 | 22321 | IslandPath-DIMOB | 2233143 | 2234807 | 1 | Pyridine nucleotide-disulfide oxidoreductase; NADH dehydrogenase (EC 1.6.99.3) |
| 2218766 | 2241087 | 22321 | IslandPath-DIMOB | 2234804 | 2235118 | 1 | Arsenical resistance operon repressor |
| 2218766 | 2241087 | 22321 | IslandPath-DIMOB | 2235118 | 2236410 | 1 | Arsenic efflux pump protein |
| 2218766 | 2241087 | 22321 | IslandPath-DIMOB | 2236429 | 2236824 | 1 | Arsenate reductase (EC 1.20.4.1) |
| 2218766 | 2241087 | 22321 | IslandPath-DIMOB | 2237030 | 2237512 | -1 | DUF1541 domain-containing protein |
| 2218766 | 2241087 | 22321 | IslandPath-DIMOB | 2237532 | 2238965 | -1 | Multicopper oxidase |
| 2218766 | 2241087 | 22321 | IslandPath-DIMOB | 2239218 | 2239391 | -1 | hypothetical protein |
| 2218766 | 2241087 | 22321 | IslandPath-DIMOB | 2240072 | 2240803 | -1 | FIG003846: hypothetical protein |
| 2218766 | 2241087 | 22321 | IslandPath-DIMOB | 2240827 | 2241087 | -1 | FIG007303: uncharacterized protein |
| 2256537 | 2263846 | 7309 | SIGI-HMM | 2256537 | 2256680 | -1 | Type I restriction-modification system, restriction subunit R (EC 3.1.21.3) |

| | | | | | | | |
|---|---|---|---|---|---|---|---|
| 2256537 | 2263846 | 7309 | SIGI-HMM | 2256861 | 2257346 | -1 | Acetyltransferase, GNAT family |
| 2256537 | 2263846 | 7309 | SIGI-HMM | 2257599 | 2258078 | -1 | LSU m3Psi1915 methyltransferase RlmH |
| 2256537 | 2263846 | 7309 | SIGI-HMM | 2258489 | 2259280 | -1 | Zn-dependent hydrolase (beta-lactamase superfamily) |
| 2256537 | 2263846 | 7309 | SIGI-HMM | 2259895 | 2260683 | -1 | hypothetical protein SA_21 |
| 2256537 | 2263846 | 7309 | SIGI-HMM | 2260684 | 2262021 | -1 | FIG011501: YycH protein |
| 2256537 | 2263846 | 7309 | SIGI-HMM | 2262014 | 2263846 | -1 | Two-component sensor kinase SA14-24 |
| 2334631 | 2346680 | 12049 | IslandPath-DIMOB | 2334631 | 2335446 | -1 | Phage lysin, glycosyl hydrolase, family 25 |
| 2334631 | 2346680 | 12049 | IslandPath-DIMOB | 2336069 | 2337229 | 1 | Cysteine desulfurase (EC 2.8.1.7), SufS subfamily |
| 2334631 | 2346680 | 12049 | IslandPath-DIMOB | 2337233 | 2337910 | 1 | YcfA protein |
| 2334631 | 2346680 | 12049 | IslandPath-DIMOB | 2337923 | 2338306 | 1 | hypothetical protein |
| 2334631 | 2346680 | 12049 | IslandPath-DIMOB | 2338306 | 2339196 | 1 | hypothetical protein |
| 2334631 | 2346680 | 12049 | IslandPath-DIMOB | 2339403 | 2339609 | 1 | hypothetical protein |
| 2334631 | 2346680 | 12049 | IslandPath-DIMOB | 2339613 | 2340359 | 1 | 2-haloalkanoic acid dehalogenase (EC 3.8.1.2) |
| 2334631 | 2346680 | 12049 | IslandPath-DIMOB | 2340773 | 2341069 | 1 | SSU ribosomal protein S6p |
| 2334631 | 2346680 | 12049 | IslandPath-DIMOB | 2341091 | 2341600 | 1 | Single-stranded DNA-binding protein |
| 2334631 | 2346680 | 12049 | IslandPath-DIMOB | 2341647 | 2341889 | 1 | SSU ribosomal protein S18p @ SSU ribosomal protein S18p, zinc-independent |
| 2334631 | 2346680 | 12049 | IslandPath-DIMOB | 2342218 | 2342472 | 1 | FIG01109292: hypothetical protein |
| 2334631 | 2346680 | 12049 | IslandPath-DIMOB | 2342609 | 2343172 | 1 | Phage protein |
| 2334631 | 2346680 | 12049 | IslandPath- | 2343256 | 2343609 | 1 | FIG01108454: hypothetical protein |

| | | | | | | | |
|---|---|---|---|---|---|---|---|
| | | | DIMOB | | | | |
| 2334631 | 2346680 | 12049 | IslandPath-DIMOB | 2343680 | 2343937 | -1 | FIG01107864: hypothetical protein |
| 2334631 | 2346680 | 12049 | IslandPath-DIMOB | 2344094 | 2344843 | 1 | (2-pyrone-4,6-)dicarboxylic acid hydrolase |
| 2334631 | 2346680 | 12049 | IslandPath-DIMOB | 2344847 | 2345107 | -1 | hypothetical protein |
| 2334631 | 2346680 | 12049 | IslandPath-DIMOB | 2345409 | 2345663 | 1 | FIG01108348: hypothetical protein |
| 2334631 | 2346680 | 12049 | IslandPath-DIMOB | 2345826 | 2346680 | 1 | hypothetical protein |
| 2413079 | 2420464 | 7385 | IslandPath-DIMOB | 2413079 | 2413753 | 1 | Mobile element protein |
| 2413079 | 2420464 | 7385 | IslandPath-DIMOB | 2414494 | 2414979 | -1 | immunodominant antigen B |
| 2413079 | 2420464 | 7385 | IslandPath-DIMOB | 2415605 | 2416207 | 1 | short chain dehydrogenase |
| 2413079 | 2420464 | 7385 | IslandPath-DIMOB | 2416353 | 2416958 | -1 | hypothetical protein |
| 2413079 | 2420464 | 7385 | IslandPath-DIMOB | 2417444 | 2418253 | -1 | CAAX amino terminal protease family |
| 2413079 | 2420464 | 7385 | IslandPath-DIMOB | 2418278 | 2418478 | -1 | hypothetical protein |
| 2413079 | 2420464 | 7385 | IslandPath-DIMOB | 2419035 | 2419253 | 1 | hypothetical protein |
| 2413079 | 2420464 | 7385 | IslandPath-DIMOB | 2419790 | 2420464 | -1 | Mobile element protein |
| 2468992 | 2475299 | 6307 | IslandPath-DIMOB | 2468992 | 2469786 | 1 | Chromosome (plasmid) partitioning protein ParA / Sporulation initiation inhibitor protein Soj |
| 2468992 | 2475299 | 6307 | IslandPath-DIMOB | 2469791 | 2469991 | 1 | FIG01110712: hypothetical protein |

| | | | | | | | |
|---|---|---|---|---|---|---|---|
| 2468992 | 2475299 | 6307 | IslandPath-DIMOB | 2470516 | 2470686 | 1 | hypothetical protein |
| 2468992 | 2475299 | 6307 | IslandPath-DIMOB | 2470829 | 2471371 | 1 | Mobile element protein |
| 2468992 | 2475299 | 6307 | IslandPath-DIMOB | 2471495 | 2471761 | -1 | hypothetical protein |
| 2468992 | 2475299 | 6307 | IslandPath-DIMOB | 2471886 | 2472374 | 1 | Hypothetical protein, ydbS homolog |
| 2468992 | 2475299 | 6307 | IslandPath-DIMOB | 2472382 | 2473788 | 1 | Hypothetical protein, ydbT homolog (Epidermicin-like peptide) |
| 2468992 | 2475299 | 6307 | IslandPath-DIMOB | 2473874 | 2474029 | 1 | hypothetical protein |
| 2468992 | 2475299 | 6307 | IslandPath-DIMOB | 2474074 | 2474364 | -1 | hypothetical protein |
| 2468992 | 2475299 | 6307 | IslandPath-DIMOB | 2474368 | 2474643 | -1 | hypothetical protein |
| 2468992 | 2475299 | 6307 | IslandPath-DIMOB | 2474730 | 2475299 | -1 | hypothetical protein |

**Table S2. Subsystem features of *Staphylococcus* spp.**

| Subsystem Features | *S.capitis* TE8 | *S.capitis* AYP1020 | *S.caprae* M23864 | *S.epidermidis* ATCC12228 |
|---|---|---|---|---|
| **Cofactors, Vitamins, Prosthetic Groups, Pigments** | **143** | **148** | **148** | **181** |
| Biotin | 9 | 6 | 6 | 29 |
| Thiamin biosynthesis | 13 | 12 | 13 | 16 |
| Quinone cofactors | 15 | 14 | 14 | 14 |
| Tetrapyrroles | 16 | 17 | 18 | 17 |
| Riboflavin, FMN, FAD | 13 | 14 | 13 | 13 |
| Pyridoxine | 6 | 8 | 8 | 9 |
| NAD and NADP | 6 | 6 | 6 | 10 |
| **Folate and pterines :** | **47** | **53** | **51** | **55** |
| Molybdenum cofactor biosynthesis | 14 | 14 | 14 | 14 |
| Folate Biosynthesis | 13 | 21 | 8 | 19 |
| 5-FCL-like protein | 20 | 18 | 17 | 22 |
| Lipoic acid metabolism | 4 | 4 | 4 | 4 |
| Coenzyme A | 14 | 14 | 15 | 14 |

| | | | | |
|---|---|---|---|---|
| **Cell Wall and Capsule** | **91** | **93** | **92** | **91** |
| Capsular and extracellular polysacchrides | 16 | 11 | 18 | 10 |
| Murein Hydrolases | 2 | 2 | 2 | 3 |
| Peptidoglycan Biosynthesis | 25 | 29 | 25 | 26 |
| UDP-N-acetylmuramate from Fructose-6-phosphate Biosynthesis | 10 | 9 | 9 | 9 |
| tRNA-dependent amino acid transfers | 5 | 5 | 6 | 5 |
| YjeE | - | 3 | 3 | 3 |
| Recycling of Peptidoglycan Amino Sugars | 1 | - | - | - |
| Recycling of Peptidoglycan Amino Acids | 1 | 1 | 1 | 1 |
| Teichoic and lipoteichoic acids biosynthesis | 19 | 22 | 18 | 26 |
| D-Alanyl Lipoteichoic Acid Biosynthesis | 4 | 4 | 4 | 4 |
| Sortase | 7 | 6 | 5 | 3 |
| Polyglycerolphosphate lipoteichoic acid biosynthesis | 1 | 1 | 1 | 1 |
| **Virulence, Disease and Defense** | **81** | **68** | **73** | **84** |
| Adhesins in Staphylococcus | 14 | 13 | 7 | 8 |
| Streptococcus pyogenes recombinatorial zone | 1 | 1 | 1 | 1 |

| | | | | |
|---|---|---|---|---|
| Bacitracin Stress Response | 8 | 8 | 8 | 8 |
| Colicin V and Bacteriocin Production Cluster | 6 | 6 | 6 | 6 |
| **Resistance to antibiotics and toxic compounds :** | **43** | **31** | **42** | **52** |
| Copper homeostasis | 4 | 3 | 2 | 7 |
| Bile hydrolysis | 3 | 2 | 2 | 2 |
| Cobalt-zinc-cadmium resistance | 6 | 4 | 5 | 6 |
| Multidrug Resistance, 2-protein version Found in Gram-positive bacteria | 3 | 3 | 4 | 3 |
| Mercuric reductase | 2 | 2 | 4 | 3 |
| Mercury resistance operon | 1 | 1 | 2 | 3 |
| Teicoplanin-resistance in Staphylococcus | 3 | 3 | 3 | 3 |
| Aminoglycoside adenylyltransferases | 1 | - | 4 | - |
| Resistance to fluoroquinolones | 4 | 4 | 4 | 4 |
| Arsenic resistance | 9 | 8 | 7 | 10 |
| Fosfomycin resistance | 1 | - | 1 | 1 |
| Beta-lactamase | 4 | 1 | 4 | 5 |
| Cadmium resistance | 2 | - | 2 | 5 |

| | | | | |
|---|---|---|---|---|
| Multidrug Resistance Efflux Pumps | -- | - | 2 | - |
| **Invasion and intracellular resistance** | **9** | **9** | **9** | **9** |
| **Potassium metabolism** | **9** | **9** | **9** | **5** |
| Potassium homeostasis | 9 | 9 | 9 | 5 |
| **Miscellaneous** | **26** | **39** | **32** | **38** |
| Iron-sulfur cluster assembly | 13 | 13 | 12 | 12 |
| Scaffold proteins for [4Fe-4S] cluster assembly (MRP family) | - | 7 | 7 | 7 |
| Single-Rhodanese-domain proteins | 1 | 1 | 1 | 1 |
| Conserved gene cluster possibly involved in RNA metabolism | 5 | 5 | 5 | 5 |
| Phosphoglycerate mutase protein family | 3 | 3 | 3 | 4 |
| Muconate lactonizing enzyme family | 1 | 7 | 1 | 6 |
| Broadly distributed proteins not in subsystems | 3 | 3 | 3 | 3 |
| **Phages, Prophages, Transposable elements, Plasmids** | **13** | **15** | **18** | **17** |
| Transposable elements (Tn552) | - | - | - | 12 |
| Phage tail proteins | 2 | 2 | 3 | - |
| Phage replication | 4 | - | 3 | - |

| | | | | |
|---|---|---|---|---|
| Phage packaging machinery | 2 | 4 | 1 | - |
| Phage tail proteins 2 | 1 | 1 | 3 | - |
| Phage lysis modules | 2 | 2 | 2 | - |
| Phage capsid proteins | 1 | 4 | 2 | 2 |
| Phage introns | - | - | 1 | - |
| Listeria Pathogenicity Island LIPI-1 extended | 1 | 1 | 1 | 1 |
| Rolling-circle replication | - | - | 1 | 2 |
| **Membrane Transport** | **34** | **37** | **40** | **53** |
| **ABC transporters:** | **11** | **11** | **13** | **15** |
| ABC transporter alkylphosphonate | 4 | 4 | 4 | 4 |
| ABC transporter oligopeptide | 6 | 6 | 8 | 9 |
| ABC transporter dipeptide | 1 | 1 | 1 | 2 |
| **Protein translocation across cytoplasmic membrane:** | **16** | **16** | **9** | **14** |
| Bacterial signal recognition particle (SRP) | 3 | 3 | 3 | 3 |
| SecY2-SecA2 Specialized Transport System | 2 | 7 | - | 7 |
| EcsAB transporter affecting expression and secretion of secretory preproteins | 4 | 4 | 4 | 4 |

| | | | | |
|---|---|---|---|---|
| Twin-arginine translocation system | 2 | 2 | 2 | - |
| **Cation transporters:** | **6** | **9** | **15** | **9** |
| Magnesium transport | 3 | 5 | 5 | 5 |
| Copper Transport System | 3 | 4 | 3 | 4 |
| Transport of Nickel and Cobalt | - | - | 7 | - |
| **Uni- Sym- and Antiporters:** | **1** | **1** | **1** | **15** |
| Proton-dependent Peptide Transporters | 1 | 1 | 1 | 1 |
| Multi-subunit cation antiporter | - | - | - | 14 |
| Ton and Tol transport systems | - | - | 2 | - |
| **Iron acquisition and metabolism** | **22** | **22** | **37** | **27** |
| Siderophore Anthrachelin | - | - | 4 | 4 |
| **Iron acquisition and metabolism** | **22** | **22** | **33** | **23** |
| Heme, hemin uptake and utilization systems in GramPositives | 19 | 19 | 23 | 17 |
| Ferrous iron transporter EfeUOB, low-pH-induced) | 3 | 3 | 4 | - |
| Iron acquisition in Streptococcus | **-** | **-** | 5 | 5 |
| Hemin transport system | **-** | **-** | 1 | 1 |
| **RNA Metabolism** | **107** | **121** | **121** | **117** |

| | | | | |
|---|---|---|---|---|
| **RNA processing and modification** | **89** | **101** | **101** | **98** |
| RNA pseudouridine syntheses | 7 | 7 | 7 | 7 |
| tRNA nucleotidyltransferase | 1 | 1 | 1 | 1 |
| Methylthiotransferases | 1 | 2 | 2 | 2 |
| Ribonucleases in Bacillus | 3 | 5 | 5 | 5 |
| RNA processing and degradation, bacterial | 2 | 3 | 4 | 3 |
| RNA methylation | 11 | 15 | 14 | 16 |
| 16S rRNA modification within P site of ribosome | 8 | 8 | 8 | 8 |
| tRNA modification Bacteria | 27 | 28 | 29 | 27 |
| mnm5U34 biosynthesis bacteria | 6 | 6 | 5 | 5 |
| Queuosine-Archaeosine Biosynthesis | 11 | 14 | 13 | 12 |
| Ribonuclease H | 2 | 3 | 3 | 3 |
| RNA processing orphans | 1 | 1 | 1 | 1 |
| tRNA processing | 6 | 8 | 9 | 8 |
| **Transcription:** | **18** | **20** | **20** | **19** |
| Transcription initiation, bacterial sigma factors | 3 | 4 | 3 | 3 |
| RNA polymerase bacterial | 5 | 5 | 5 | 5 |

| | | | | |
|---|---|---|---|---|
| Transcription factors bacterial | 9 | 10 | 10 | 10 |
| Rrf2 family transcriptional regulators | 1 | 1 | 2 | 1 |
| **Nucleosides and Nucleotides** | **88** | **87** | **87** | **91** |
| **Pyrimidines** | **28** | **30** | **30** | **27** |
| pyrimidine conversions | 17 | 19 | 19 | 16 |
| De Novo Pyrimidine Synthesis | 11 | 11 | 11 | 11 |
| **Purines** | **42** | **41** | **39** | **44** |
| De Novo Purine Biosynthesis | 16 | 15 | 15 | 15 |
| Xanthine Metabolism in Bacteria | 3 | 3 | 3 | 3 |
| Purine conversions | 23 | 23 | 21 | 24 |
| **Nucleosides and Nucleotides** | **10** | **10** | **10** | **11** |
| Ribonucleotide reduction | 7 | 7 | 7 | 7 |
| Adenosyl nucleosidases | 3 | 3 | 3 | 3 |
| **Detoxification:** | **8** | **6** | **8** | **9** |
| Nucleoside triphosphate pyrophosphohydrolase MazG | 1 | 1 | 1 | 1 |
| Nudix proteins (nucleoside triphosphate hydrolases) | 2 | 1 | 2 | 4 |
| Housecleaning nucleoside triphosphate pyrophosphatases | 5 | 4 | 5 | 4 |

| | | | | |
|---|---|---|---|---|
| **Protein Metabolism** | **165** | **199** | **184** | **207** |
| **Protein folding** | 11 | 11 | 11 | 11 |
| GroEL GroES | 6 | 6 | 6 | 6 |
| Protein chaperones | 4 | 4 | 4 | 4 |
| Peptidyl-prolyl cis-trans isomerase | 1 | 1 | 1 | 1 |
| **Protein biosynthesis** | **112** | **144** | **125** | **149** |
| tRNA aminoacylation, Val | 1 | 1 | 1 | 1 |
| tRNA aminoacylation, Met | 1 | 1 | 1 | 1 |
| tRNA aminoacylation, Ile | 1 | 1 | 1 | 1 |
| tRNA aminoacylation, Arg | 1 | 1 | 1 | 1 |
| Translation initiation factors bacterial | 5 | 5 | 6 | 5 |
| Ribosome SSU bacterial | 25 | 25 | | 26 |
| tRNA aminoacylation, Gly | 1 | 1 | 1 | 1 |
| tRNA aminoacylation, Ala | 1 | 1 | 1 | 1 |
| Ribosome activity modulation | 1 | 1 | 1 | 1 |
| tRNA aminoacylation, Trp | 1 | 1 | 1 | 1 |

| | | | | |
|---|---|---|---|---|
| Ribosome biogenesis bacterial | - | 12 | 13 | 13 |
| Ribosome LSU bacterial | 39 | 37 | 39 | 40 |
| Programmed frameshift | 2 | 2 | 2 | 2 |
| tRNA aminoacylation, Cys | 1 | 1 | 1 | 2 |
| Translation termination factors bacterial | 9 | 10 | 10 | 10 |
| Universal GTPases | - | 17 | 18 | 17 |
| tRNA aminoacylation, His | 1 | 1 | 1 | 1 |
| tRNA aminoacylation, Asp and Asn | 5 | 5 | 5 | 5 |
| Translation elongation factors bacterial | 5 | 5 | 5 | 1 |
| tRNA aminoacylation, Lys | 1 | 1 | 1 | 1 |
| tRNA aminoacylation, Thr | 1 | 1 | 1 | 1 |
| tRNA aminoacylation, Pro | - | 2 | 2 | 2 |
| Translation elongation factor G family | 1 | 1 | 1 | 1 |
| tRNA aminoacylation, Glu and Gln | 5 | 5 | 5 | 5 |
| tRNA aminoacylation, Ser | 1 | 1 | 1 | 1 |
| tRNA aminoacylation, Tyr | 1 | 1 | 1 | 1 |
| tRNA aminoacylation, Leu | 1 | 1 | 2 | 1 |

| | | | | |
|---|---|---|---|---|
| tRNA aminoacylation, Phe | 3 | 3 | 3 | 3 |
| **Protein processing and modification:** | **21** | **21** | **25** | **24** |
| Lipoprotein Biosynthesis | 2 | 2 | 2 | 3 |
| Signal peptidase | 4 | 4 | 4 | 5 |
| G3E family of P-loop GTPases | 9 | 9 | 9 | 10 |
| Protein deglycation | 2 | 2 | 2 | 2 |
| Peptide methionine sulfoxide reductase | 4 | 4 | 4 | 4 |
| N-linked Glycosylation in Bacteria | - | - | 4 | - |
| **Protein degradation** | **21** | **23** | **23** | **23** |
| Aminopeptidases (EC 3.4.11.-) | 2 | 2 | 2 | 2 |
| Protein degradation | 2 | 2 | 1 | 1 |
| Metallocarboxypeptidases (EC 3.4.17.-) | 1 | 1 | 1 | 2 |
| Proteasome bacterial | 5 | 5 | 5 | 5 |
| Serine endopeptidase (EC 3.4.21.-) | - | - | 1 | 1 |
| Proteolysis in bacteria, ATP-dependent | 10 | 12 | 11 | 11 |
| Omega peptidases (EC 3.4.19.-) | 1 | 1 | 1 | 1 |
| **Cell Division and Cell Cycle** | **19** | **41** | **40** | **39** |

| | | | | |
|---|---|---|---|---|
| **Checkpoint control** | - | 5 | 5 | 5 |
| **Cell Division and Cell Cycle** | **19** | **36** | **35** | **34** |
| Control of cell elongation - division cycle in Bacilli | - | 10 | 10 | 10 |
| YgjD and YeaZ | - | 2 | 2 | 2 |
| Macromolecular synthesis operon | - | 7 | 6 | 6 |
| Bacterial Cytoskeleton | 19 | 17 | 17 | 16 |
| **Regulation and Cell signaling** | **49** | **54** | **49** | **37** |
| HPr catabolite repression system | 3 | 2 | 2 | 2 |
| cAMP signaling in bacteria | 3 | 3 | 4 | - |
| LysR-family proteins in Salmonella enterica Typhimurium | 1 | 1 | 2 | - |
| LysR-family proteins in Escherichia coli | 1 | 2 | 2 | - |
| Sex pheromones in Enterococcus faecalis and other Firmicutes | 6 | 6 | 7 | 7 |
| Stringent Response, (p)ppGpp metabolism | 1 | 1 | 1 | 1 |
| Cell envelope-associated LytR-CpsA-Psr transcriptional attenuators | 3 | 3 | 4 | 3 |
| Biofilm formation in Staphylococcus | 15 | 16 | 11 | 6 |

| | | | | |
|---|---|---|---|---|
| Regulation of virulence | 3 | 3 | 3 | 3 |
| **Programmed Cell Death and Toxin-antitoxin Systems** | **13** | **17** | **15** | **15** |
| MazEF toxin-antitoxing (programmed cell death) system | 2 | 3 | 2 | 2 |
| Murein hydrolase regulation and cell death | 9 | 9 | 9 | 11 |
| Toxin-antitoxin replicon stabilization systems | - | 2 | 2 | - |
| Phd-Doc, YdcE-YdcD toxin-antitoxin (programmed cell death) systems | 2 | 3 | 2 | 2 |
| **Secondary Metabolism** | **6** | **6** | **4** | **5** |
| Lanthionine Synthetases | 2 | 2 | - | 1 |
| Auxin biosynthesis | 4 | 4 | 4 | 4 |
| **DNA Metabolism** | **125** | **138** | **105** | **92** |
| **DNA repair** | **55** | **59** | **58** | **54** |
| Uracil-DNA glycosylase | 1 | 1 | 1 | 1 |
| DNA repair, bacterial MutL-MutS system | 4 | 4 | 4 | 4 |
| DNA repair, UvrABC system | 5 | 4 | 4 | 5 |
| DNA repair, bacterial photolyase | 1 | 1 | 1 | 1 |
| DNA repair, bacterial DinG and relatives | 1 | 1 | 1 | 1 |

| | | | | |
|---|---|---|---|---|
| 2-phosphoglycolate salvage | 2 | 3 | 2 | 1 |
| DNA repair system including RecA, MutS and a hypothetical protein | 2 | 2 | 2 | 2 |
| ATP-dependent Nuclease | 2 | 2 | 2 | 2 |
| DNA repair, bacterial | 17 | 18 | 19 | 16 |
| DNA repair, bacterial RecFOR pathway | 8 | 11 | 10 | 10 |
| DNA repair, bacterial RecBCD pathway | 1 | 1 | 1 | 1 |
| DNA repair, bacterial UvrD and related helicases | 1 | 1 | 1 | 1 |
| DNA Repair Base Excision | 10 | 10 | 10 | 9 |
| YcfH | 1 | 1 | 1 | 1 |
| Type I Restriction-Modification | 5 | 9 | 5 | - |
| Restriction-Modification System | 5 | 9 | 5 | - |
| DNA structural proteins, bacterial | 2 | 2 | 2 | 2 |
| **DNA replication:** | **30** | **32** | **7** | **30** |
| DNA topoisomerases, Type I, ATP-independent | 2 | 3 | 2 | 3 |
| DNA replication strays | 1 | 1 | 1 | 2 |
| DNA-replication | 23 | 24 | - | 21 |

| | | | | |
|---|---|---|---|---|
| DNA topoisomerases, Type II, ATP-dependent | 4 | 4 | 4 | 4 |
| **DNA uptake, competence** | **27** | **26** | **27** | **5** |
| DNA processing cluster | 4 | 5 | 4 | 5 |
| Late competence | 13 | 12 | 13 | - |
| Gram Positive Competence | 10 | 9 | 10 | - |
| **Fatty Acids, Lipids, and Isoprenoids** | **70** | **89** | **89** | **92** |
| Cardiolipin synthesis | 2 | 2 | 2 | 2 |
| Glycerolipid and Glycerophospholipid Metabolism in Bacteria | 21 | 21 | 21 | 23 |
| Triacylglycerol metabolism | 8 | 8 | 8 | 8 |
| **Fatty acids** | **24** | **22** | **21** | **21** |
| Fatty Acid Biosynthesis FASII | 17 | 18 | 17 | 16 |
| Fatty acid metabolism cluster | 7 | 4 | 4 | 5 |
| Polyhydroxybutyrate metabolism | 8 | - | - | 7 |
| **Isoprenoids** | **7** | **36** | **37** | **31** |
| Carotenoids | - | 3 | 3 | - |
| Mevalonate Branch of Isoprenoid Biosynthesis | 7 | 7 | 7 | 7 |

| | | | | |
|---|---|---|---|---|
| Isoprenoinds for Quinones | - | 4 | 4 | 4 |
| Isoprenoid Biosynthesis | - | 12 | 12 | 13 |
| Polyprenyl Diphosphate Biosynthesis | - | 7 | 8 | 4 |
| Isoprenoid Biosynthesis: Interconversions | - | 3 | 3 | 3 |
| **Nitrogen Metabolism** | **21** | **23** | **23** | **26** |
| Nitric oxide synthase | 2 | 2 | 2 | 2 |
| Nitrosative stress | - | - | - | 1 |
| Nitrate and nitrite ammonification | 8 | 8 | 9 | 8 |
| Ammonia assimilation | 5 | 7 | 6 | 9 |
| Denitrifying reductase gene clusters | 6 | 6 | 6 | 6 |
| **Dormancy and Sporulation** | **4** | **4** | **4** | **10** |
| Sporulation-associated proteins with broader functions | 3 | -3 | 3 | 3 |
| Persister Cells | 1 | 1 | 1 | - |
| Sporulation Cluster | - | - | - | 7 |
| **Respiration** | **32** | **30** | **39** | **31** |
| Biotin | 9 | 6 | 6 | 29 |
| **Electron accepting reactions :** | **14** | **14** | **22** | **16** |

| | | | | |
|---|---|---|---|---|
| Terminal cytochrome d ubiquinol oxidases | 4 | 4 | 8 | 4 |
| Terminal cytochrome C oxidases | 4 | 4 | 4 | 4 |
| Anaerobic respiratory reductases | 2 | 2 | 2 | 4 |
| Terminal cytochrome oxidases | 4 | 4 | 8 | 4 |
| **Electron donating reactions :** | **8:** | **7:** | **8:** | **9:** |
| Respiratory dehydrogenases 1 | 5 | 4 | 5 | 5 |
| Succinate dehydrogenase | 3 | 3 | 3 | 4 |
| Quinone oxidoreductase family | 1 | 1 | 1 | 1 |
| Biogenesis of cytochrome c oxidases | 2 | 2 | 2 | 2 |
| Formate hydrogenase | 5 | 4 | 4 | 2 |
| Soluble cytochromes and functionally related electron carriers | 2 | 2 | 2 | 1 |
| **Stress Response** | **68** | **71** | **68** | **73** |
| **Osmotic stress :** | **17** | **15** | **12** | **16** |
| Osmoregulation | 1 | 1 | 1 | 1 |
| Choline and Betaine Uptake and Betaine Biosynthesis | 16 | 14 | 11 | 15 |
| **Oxidative stress** | **27** | **30** | **30** | **29** |
| NADPH:quinone oxidoreductase 2 | - | 1 | - | - |

| | | | | |
|---|---|---|---|---|
| Glutathione: Non-redox reactions | 1 | 1 | 1 | 1 |
| CoA disulfide thiol-disulfide redox system | 1 | 1 | 2 | 1 |
| Redox-dependent regulation of nucleus processes | 5 | 5 | 5 | 5 |
| Glutathione: Redox cycle | 1 | 1 | 1 | 1 |
| Glutaredoxins | 2 | 2 | 2 | 2 |
| Oxidative stress | 14 | 14 | 14 | 14 |
| Protection from Reactive Oxygen Species | 3 | 3 | 3 | 3 |
| Cluster containing Glutathione synthetase | - | 2 | 2 | 2 |
| **Cold shock, CspA family of proteins** | **2** | **2** | **2** | **2** |
| **Heat shock dnaK gene cluster extended** | **13** | **15** | **15** | **16** |
| Detoxification | 9 | 6 | 8 | 9 |
| Flavohaemoglobin | 2 | 2 | 2 | 1 |
| SigmaB stress responce regulation | 2 | 3 | 3 | 4 |
| Bacterial hemoglobins | 2 | 2 | 2 | 2 |
| Hfl operon | 2 | 2 | 2 | 2 |
| Periplasmic Stress Response | - | - | - | 1 |
| **Metabolism of Aromatic Compounds** | **6** | **7** | **5** | **5** |

| | | | | |
|---|---|---|---|---|
| **Peripheral pathways for catabolism of aromatic compounds :** | **2** | **2** | **2** | **2** |
| Salicylate ester degradation | 1 | 1 | 1 | 1 |
| Quinate degradation | 1 | 1 | 1 | 1 |
| Catechol branch of beta-ketoadipate pathway | 2 | 2 | - | - |
| Salicylate and gentisate catabolism | 2 | 2 | 2 | 2 |
| Gentisate degradation | - | 1 | 1 | 1 |
| **Amino Acids and Derivatives** | **262** | **278** | **315** | **335** |
| **Glutamine, glutamate, aspartate, asparagine; ammonia assimilation** | **16** | **18** | **17** | **21** |
| Glutamine, Glutamate, Aspartate and Asparagine Biosynthesis | 8 | 10 | 9 | 12 |
| Glutamate dehydrogenases | 1 | 1 | 1 | 1 |
| Glutamine synthetases | 1 | 1 | 1 | 1 |
| Poly-gamma-glutamate biosynthesis | 6 | 6 | 6 | 7 |
| **Histidine Metabolism** | **5** | **5** | **17** | **11** |
| Histidine Degradation | 5 | 5 | 5 | 5 |
| Histidine Biosynthesis | - | - | 12 | 11 |

| | | | | |
|---|---|---|---|---|
| **Arginine; urea cycle, polyamines :** | **59** | **59** | **87** | **105** |
| Polyamine Metabolism | 13 | 13 | 13 | 15 |
| Arginine and Ornithine Degradation | 14 | 14 | 14 | 19 |
| Arginine Biosynthesis -- gjo | - | - | 14 | 17 |
| Urea carboxylase and Allophanate hydrolase cluster | 7 | 7 | 7 | 7 |
| Arginine Deiminase Pathway | 10 | 10 | 10 | 15 |
| Urease subunits | 7 | 7 | 7 | 7 |
| Arginine Biosynthesis extended | - | - | 14 | 17 |
| Urea decomposition | 8 | 8 | 8 | 8 |
| **Lysine, threonine, methionine, and cysteine** | **64** | **76** | **76** | **77** |
| Methionine Biosynthesis | 21 | 21 | 21 | 21 |
| Threonine degradation | 4 | 3 | 3 | 4 |
| Lysine Biosynthesis DAP Pathway, GJO scratch | 9 | 11 | 11 | 11 |
| Methionine Degradation | 12 | 12 | 12 | 12 |
| Threonine and Homoserine Biosynthesis | 7 | 7 | 7 | 7 |
| Cysteine Biosynthesis | 9 | 9 | 9 | 9 |
| Lysine Biosynthesis DAP Pathway | - | 11 | 11 | 11 |

| | | | | |
|---|---|---|---|---|
| Lysine degradation | 2 | 2 | 2 | 2 |
| **Branched-chain amino acids:** | **32** | **30** | **30** | **33** |
| Isoleucine degradation | 9 | 8 | 8 | 10 |
| Branched-Chain Amino Acid Biosynthesis | 11 | 11 | 11 | 10 |
| Valine degradation | 7 | 6 | 6 | 8 |
| Leucine Biosynthesis | 5 | 5 | 5 | 5 |
| **Aromatic amino acids and derivatives** | **42** | **44** | **43** | **42** |
| Common Pathway For Synthesis of Aromatic Compounds (DAHP synthase to chorismate) | 7 | 7 | 7 | 7 |
| Chorismate Synthesis | 10 | 10 | 10 | 10 |
| Chorismate: Intermediate for synthesis of Tryptophan, PAPA antibiotics, PABA, 3-hydroxyanthranilate and more. | 11 | 12 | 12 | 11 |
| Phenylalanine and Tyrosine Branches from Chorismate | 4 | 4 | 4 | 4 |
| Tryptophan synthesis | 10 | 11 | 10 | 10 |
| **Proline and 4-hydroxyproline** | **6** | **7** | **7** | **6** |
| Proline Synthesis | 1 | 1 | 1 | 1 |
| A Hypothetical Protein Related to Proline Metabolism | 2 | 2 | 2 | 2 |

| | | | | |
|---|---|---|---|---|
| Proline, 4-hydroxyproline uptake and utilization | 3 | 4 | 4 | 3 |
| **Alanine, serine, and glycine :** | **38** | **39** | **38** | **40** |
| Glycine Biosynthesis | 4 | 3 | 3 | 3 |
| Alanine biosynthesis | 7 | 7 | 6 | 6 |
| Serine Biosynthesis | 5 | 6 | 6 | 7 |
| Glycine cleavage system | 4 | 4 | 4 | 5 |
| Glycine and Serine Utilization | 18 | 19 | 19 | 19 |
| **Sulfur Metabolism** | **20** | **13** | **12** | **19** |
| Inorganic sulfur assimilation | 8 | - | - | 8 |
| Thioredoxin-disulfide reductase | 8 | 8 | 7 | 6 |
| L-Cystine Uptake and Metabolism | 4 | 5 | 5 | 5 |
| **Phosphorus Metabolism** | **25** | **25** | **25** | **25** |
| High affinity phosphate transporter and control of PHO regulon | 8 | 8 | 8 | 8 |
| Phosphate metabolism | 15 | 15 | 15 | 15 |
| Polyphosphate | 2 | 2 | 2 | 2 |
| **Carbohydrates** | **212** | **201** | **197** | **239** |

| Central carbohydrate metabolism: | 98 | 100 | 101 | 102 |
|---|---|---|---|---|
| Methylglyoxal Metabolism | 7 | 7 | 9 | 7 |
| Pyruvate metabolism II: acetyl-CoA, acetogenesis from pyruvate | 15 | 15 | 16 | 13 |
| Pyruvate Alanine Serine Interconversions | 9 | 9 | 9 | 9 |
| Dihydroxyacetone kinases | 4 | 4 | 4 | 4 |
| Glycolysis and Gluconeogenesis | 15 | 15 | 15 | 15 |
| Dehydrogenase complexes | 10 | 10 | 10 | 14 |
| TCA Cycle | 17 | 16 | 18 | 18 |
| Pentose phosphate pathway | 8 | 8 | 8 | 11 |
| Pyruvate metabolism I: anaplerotic reactions, PEP | 5 | 7 | 4 | 4 |
| Glycolate, glyoxylate interconversions | 8 | 9 | 8 | 7 |
| **Aminosugars :** | **4** | **3** | **3** | **2** |
| Chitin and N-acetylglucosamine utilization | 4 | 3 | 3 | 2 |
| **Di- and oligosaccharides :** | **5** | **4** | **-** | **16** |
| Sucrose utilization | 5 | 4 | - | 6 |
| Lactose and Galactose Uptake and Utilization | - | - | - | 10 |

| | | | | |
|---|---|---|---|---|
| **One-carbon Metabolism :** | **34** | **29** | **28** | **29** |
| Formaldehyde assimilation: Ribulose monophosphate pathway | 6 | 1 | 2 | 3 |
| Serine-glyoxylate cycle | 23 | 23 | 21 | 21 |
| One-carbon metabolism by tetrahydropterines | 5 | 5 | 5 | 5 |
| **Organic acids :** | **7** | **7** | **7** | **7** |
| Glycerate metabolism | 5 | 5 | 5 | 5 |
| Alpha-acetolactate operon | 2 | 2 | 2 | 2 |
| **Fermentation :** | **16** | **14** | **14** | **42** |
| Butanol Biosynthesis | - | - | - | 8 |
| Fermentations: Mixed acid | - | - | - | 10 |
| Acetolactate synthase subunits | 2 | 2 | 2 | 2 |
| Fermentations: Lactate | 4 | 4 | 4 | 5 |
| Acetyl-CoA fermentation to Butyrate | 5 | 3 | 3 | 6 |
| Acetoin, butanediol metabolism | 5 | 5 | 5 | 11 |
| **Sugar alcohols :** | 16 | 15 | 15 | 11 |
| Glycerol and Glycerol-3-phosphate Uptake and Utilization | 7 | 7 | 8 | 11 |

| | | | | |
|---|---|---|---|---|
| Mannitol Utilization | 9 | 8 | 7 | - |
| **Monosaccharides :** | 32 | 29 | 29 | 30 |
| Mannose Metabolism | 7 | 5 | 5 | 4 |
| D-ribose utilization | 5 | 4 | 4 | 4 |
| Xylose utilization | 1 | 1 | 1 | 1 |
| Deoxyribose and Deoxynucleoside Catabolism | 5 | 6 | 5 | 5 |
| D-gluconate and ketogluconates metabolism | 5 | 5 | 5 | 5 |
| Fructose utilization | 9 | 8 | 9 | 11 |

**Table S3. Identity of predicted PSMβ type proteins with each other.**

| PSMβ type protein | PSMβ1 | PSMβ2 | PSMβ3 | PSMβ4 | PSMβ5 | HTP2388 |
|---|---|---|---|---|---|---|
| PSMβ1 | | | | | | 12/44 (27%) |
| PSMβ2 | 28/43 (65%) | | | | | |
| PSMβ3 | 24/43 (55%) | 34/43 (79%) | | | | |
| PSMβ4 | 25/43 (58%) | 37/44 (84%) | 35/43 (81%) | | | |
| PSMβ5 | 25/43 (58%) | 37/44 (84%) | 36/43 (83%) | 41/44 (93%) | | |
| PSMβ6 | 24/43 (55%) | 35/43 (81%) | 41/44 (93%) | 37/43 (86%) | 39/43 (90%) | |

**Table S4. Gene clusters involved in Antibiotics and Secondary metabolites synthesis using BAGEL3.**

| **Cluster** | **Type** | **Predicted product** | ***Staphylococcus capitis* TE8** | ***Staphylococcus capitis* AYP1020** | ***Staphylococcus caprae* M23864** | ***Staphylococcus epidermidis* ATCC12228** |
|---|---|---|---|---|---|---|
| 1 | Siderophore | Staphyloferrin | 75% | 75% | 75% | 75% |
| 2 | NRPS | Aureusimine | 100% | 100% | - | - |
| 3 | Lantipeptide | Epidermin | 25% | 37% | - | - |
| 4 | Terpene | Unknown | + | + | + | - |
| 5 | Microcin | Unknown | + | + | + | + |
| 6 | Microcin | Unknown | - | + | - | + |
| 7 | Microcin | Unknown | - | + | - | + |
| 8 | Microcin | Unknown | - | + | - | + |
| 9 | NRPS | Unknown | - | - | + | + |
| 10 | NRPS | Capsular polysachharide | - | - | 3% | - |
| 11 | Lantipeptide | Unknown | - | - | - | + |

**Table S5. General genome statistics of the compared genomes *Staphylococcus* spp. from RAST server.**

| **Features** | ***S. capitis* TE8** | ***S. capitis* AYP1020** | ***S. caprae* M23864** | ***S. epidermidis* ATCC 12228** |
|---|---|---|---|---|
| **Accession Number** | JMGB00000000 | GCA_001028645.1 | GCA_000160215.1 | GCA_000007645.1 |
| **Size (bp)** | 2516639 | 2443604 | 2612000 | 2499279 |
| **Isolation Source** | Human skin | Human blood | Human skin | Human blood |
| **G+C content (%)** | 32.8 | 32.9 | 33.4 | 32.1 |
| **No. of tRNA** | 56 | 63 | 50 | 61 |
| **No. of rRNA** | 4 | 19 | 4 | 16 |
| **No. of coding sequences** | 2433 | 2366 | 2492 | 2391 |
